# Design of the New *Closo*-Dodecarborate-Containing Gemcitabine Analogue for the Albumin-Based Theranostics Composition

**DOI:** 10.3390/molecules28062672

**Published:** 2023-03-15

**Authors:** Valeria I. Raskolupova, Meiling Wang, Maya A. Dymova, Gleb O. Petrov, Ivan M. Shchudlo, Sergey Yu. Taskaev, Tatyana V. Abramova, Tatyana S. Godovikova, Vladimir N. Silnikov, Tatyana V. Popova

**Affiliations:** 1 Institute of Chemical Biology and Fundamental Medicine, SB RAS, 630090 Novosibirsk, Russia; 2 Faculty of Natural Sciences, Novosibirsk State University, 630090 Novosibirsk, Russia; 3 Budker Institute of Nuclear Physics, SB RAS, 630090 Novosibirsk, Russia

**Keywords:** boron neutron capture therapy, boron delivery agents, gemcitabine analogue, boronated albumin theranostic conjugate, cell viability and proliferation, clonogenic assay

## Abstract

Combination therapy is becoming an increasingly important treatment strategy because multi-drugs can maximize therapeutic effect and overcome potential mechanisms of drug resistance. A new albumin-based theranostic containing gemcitabine *closo*-dodecaborate analogue has been developed for combining boron neutron capture therapy (BNCT) and chemotheraphy. An exo-heterocyclic amino group of gemcitabine was used to introduce *closo-*dodecaborate, and a 5′-hydroxy group was used to tether maleimide moiety through an acid-labile phosphamide linker. The *N*-trifluoroacylated homocysteine thiolactone was used to attach the gemcitabine analogue to human serum albumin (HSA) bearing Cy5 or Cy7 fluorescent dyes. The half-maximal inhibitory concentration (IC_50_) of the designed theranostic relative to T98G cells was 0.47 mM with the correlation coefficient R = 0.82. BNCT experiments resulted in a decrease in the viability of T98G cells, and the survival fraction was ≈ 0.4.

## 1. Introduction

Gemcitabine (2′,2′-difluorodeoxycytidine) has shown to be an active agent against colon, pancreatic, breast, ovary, small cell lung, head and neck, and lung cancers in amalgamation with various anticancer agents. Gemcitabine is considered a gold standard and is the first FDA approved agent used as a monotherapy in the management of advanced pancreatic cancers. However, due to its poor pharmacokinetics, there is need of a newer drug delivery system (DDS) for efficient action [1]. The major goal in DDS is to achieve a higher accumulation of the drug in tumor tissue, and thus to reduce systemic toxicity and side effects of chemotherapeutic agents. The high molecular weight of gemcitabine conjugates may be effective due to their prolonged influence on the tumor. For example, they are represented by polyethylene glycol derivatives bearing gemcitabine and folic acid residues. However, it has been found that even the high molecular weight of polyethylene glycol makes it possible to increase the half-life of conjugates in blood plasma to only two hours [2].

The use of human serum albumin (HSA) as a macromolecular carrier for therapeutic construct creation is a convenient and well-established strategy [3,4,5,6,7,8,9,10]. Albumin is a biocompatible protein and has low toxicity to the organism. Furthermore, HSA contains a number of accessible functional groups for conjugation with low molecular weight compounds, for example, with anticancer drugs. In addition, albumin is able to provide targeted transport of a therapeutic agent and its controlled release [4,5,6,7,8,9]. The reason for this is the presence of specific albumin receptors overexpressed in cancer cells (the glycoproteins Gp18, Gp30 and Gp60 and SPARC) [11,12,13,14]. The size of albumin contributes to its accumulation in the tumor due to an enhanced permeability and retention (EPR) effect.

Gemcitabine showed a weak interaction with HSA, which occurred with cooperative binding in a ratio of 1:1. The gemcitabine binding site is located between the IIA and IIB subdomain interface [15]. Noncovalent binding of gemcitabine to HSA is used in most investigations devoted to therapeutic construct creation, including nanoparticles involving gemcitabine as a therapeutic load [16,17,18,19,20]. There are also a small number of studies in which gemcitabine was covalently attached to HSA. In the study [21], the aim was to create a double-targeted delivery system for gemcitabine. It was conjugated to albumin, and the resulting construct was used to create nanoparticles equipped with folic acid as an additional target molecule. The obtained nanoparticles showed higher toxicity and a cellular uptake in MDA-MB-231 cells overexpressing folic acid receptors. At the same time, the nanoparticles remained stable during blood circulation. Other groups developed an albumin-based enzyme-sensitive (cathepsin B cleavable) nanoplatform for gemcitabine delivery [22]. In vivo NIR imaging of the resulting construct demonstrated its increased accumulation and pronounced retention in the BxPC-3 pancreatic tumors of xenografted mice. Due to reduced deactivation of gemcitabine during circulation, high accumulation and consumption in tumor tissue, and specific intracellular release of gemcitabine, excellent growth inhibition activity without side effects was achieved.

Cancers are complex and involve a variety of pathways, at the same time, tumors often have intrinsic or acquired drug resistance. Therefore, it may be insufficient for regressing tumors to use a single drug to treat cancer. As a result, combination therapy is becoming an increasingly important treatment strategy because multi-drugs can maximize the therapeutic effect and overcome potential mechanisms of drug resistance as they can modulate different signaling pathways in cancer cells. A prospective treatment method is boron neutron capture therapy (BNCT) in which drugs containing enriched boron are accumulated in tumor cells followed by their epithermal neutron beam radiation. This technique offers an advantage over conventional chemo- and radiotherapies as it selectively targets tumor cells without significantly affecting healthy tissues. A combination of gemcitabine and boron-10 for BNCT has not yet been considered by any working group, although there are enough studies in which the therapeutic conjugate contains both gemcitabine and boron atoms [23,24,25,26,27]. In particular, bortezomib, which is a first-generation proteasome inhibitor, is widely used in combination with gemcitabine [28]. In any case, the structures indicated in the abovementioned studies have no structural analogues with our design. The introduction of the boron-containing gemcitabine derivative into the HSA-based theranostic make it possible to combine BNCT and chemotherapy.

**Figure 1:**
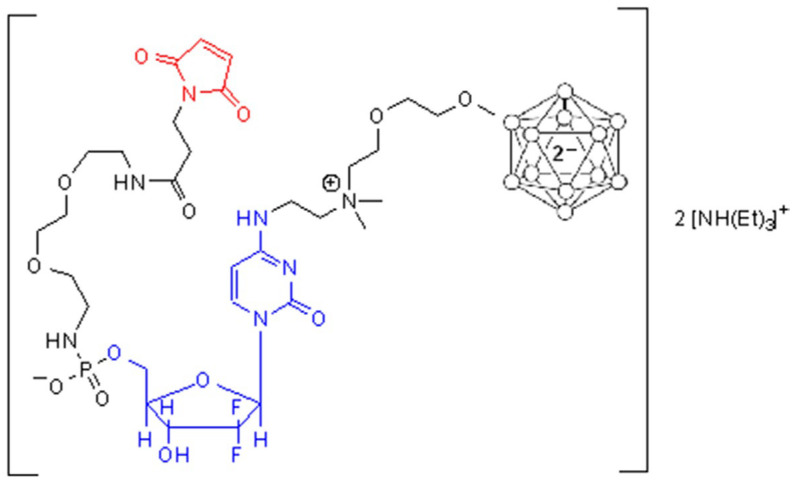
The structure of the new gemcitabine analogue containing a *closo*-dodecaborate residue (compound **1**).

In our work, we constructed a new gemcitabine analogue containing a *closo*-dodecaborate residue (B_12_H_11_) attached via an *N*-ethoxy-(2-ethoxy)-*N,N*-dimethylethan-1-aminium linker to the exocyclic group of the nitrogenous base (compound **1**, Figure 1). The 5′-hydroxyl group of gemcitabine was functionalized with a linker containing a maleimide residue at the end for connecting the gemcitabine analogue to HSA. This linker has a phosphamide bond that has been shown to be cleavable under the acidic conditions of cancer tissue [29].

Previously, we reported on the synthesis of fluorophore-labeled homocystamide conjugates of human serum albumin and their use in thiol-‘click’ chemistry [29,30,31,32,33,34,35]. Furthermore, we reported on the preparation of the novel multimodal boronated albumin-based theranostic agents, which could be accumulated in tumor cells [30]. Herein, we suggest using a boron-containing derivative of gemcitabine (compound **1**) as a part of an anticancer theranostic construct. Along with its beneficial properties as a chemotherapeutic agent, boron-containing gemcitabine derivative is a promising agent for boron-neutron capture therapy under imaging control.

## 2. Results and Discussion

### 2.1. Synthesis of Maleimide Gemcitabine Boron-Containing Derivative 1

Considering gemcitabine, a number of prodrugs have been developed. A significant number of prodrugs are obtained by modifying gemcitabine at the 5’-hydroxyl group. Conjugates with a modification at the 5’-hydroxyl group are much more stable in blood plasma and undergo metabolic transformations mainly inside the cell [36]. However, the peculiarities of the cytosine derivative’s metabolism also make it possible to use the heterocyclic base exocyclic amino group for modification [37]. The majority of effective prodrugs with improved pharmacodynamic and kinetic properties have been obtained precisely by modifying the amino group of gemcitabine. They are easy to synthesize, and a wide range of methods is available for incorporating the gemcitabine residue into conjugates with polyethylene glycol, squalene, fatty acids and other molecules. It is known that nucleoside prodrugs containing an amide bond are cleaved by cathepsins B and D, which lead to their inactivation [38,39]. It has been shown that cytosinamides are unstable in plasma, and their half-life time is several hours, which is comparable with that in the presence of cathepsins [40].

**Scheme 1:**
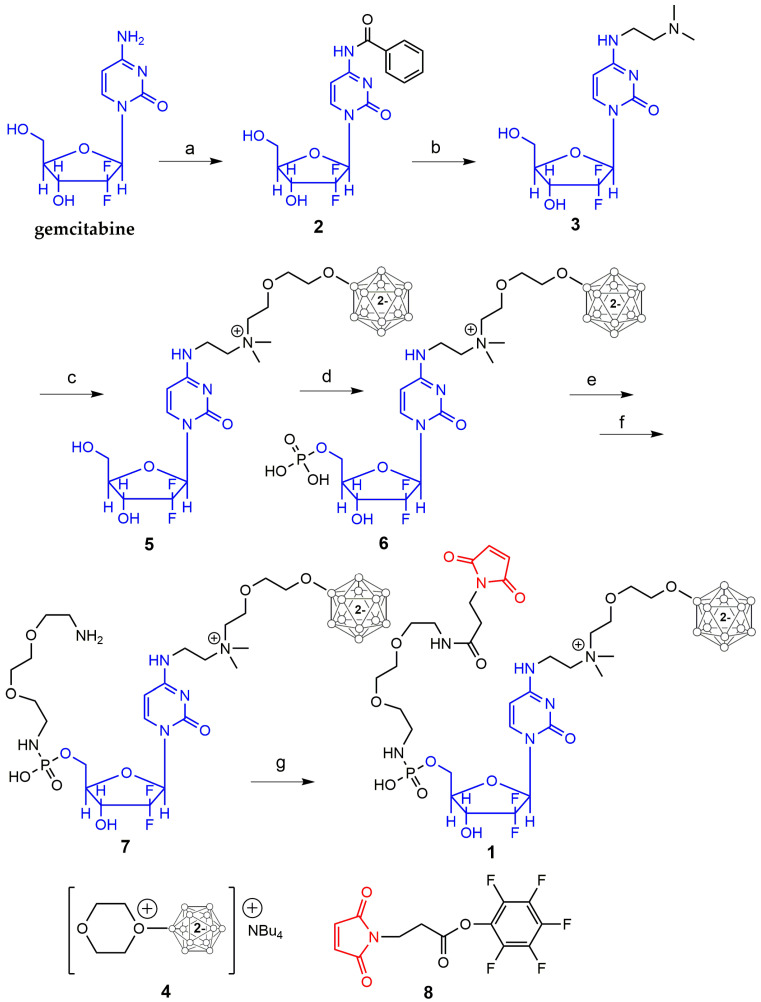
The synthesis of new HSA modifying agent **1**. Conditions: a—Bz_2_O, DMF, RT, 24 h, yield 80%; b—N,N-dimethylethylenediamine, TBD, DMF, 60 °C, 2 h, yield 33%; c—compound **4**, DMF, 70 °C, overnight, yield 92%; d—POCl_3_, proton sponge, TMP, 1.5 h, RT, yield 60%; e—PPh_3_/Py_2_S_2_, NMeI, DMSO, 30 min, RT; f—1,2-bis(2-aminoethoxy)ethane, 1 h, RT, yield 43%; g—pentafluorophenyl ester of 3-maleimidopropanoic acid **8**, TEA, DMF, 30 min, RT, yield 97%.

Our idea was to use gemcitabine as a multimodal molecule having the exo-heterocyclic amino group and 3′ and 5′ hydroxyl groups of a sugar ring. Since the 3′-OH group is likely to be important for the cytotoxic effect of gemcitabine [41,42], we modified only the 5′-OH group. We used the exo-heterocyclic amino group of gemcitabine to introduce *closo-*dodecaborate and the 5′-OH group to tether a maleimide moiety through a phosphate and extended hydrophilic diamino linker. The synthesis of this new agent is shown in Scheme 1. It is worth noting that according to the literature [43,44], gemcitabine needs to be phosphorylated for anticancer effects and the phosphamide bond of the final conjugate may be hydrolyzed under acidic conditions in lisosomes after entering cancer cells [29]. Thus, such a modification should not lead to a decrease in the cytostatic properties of gemcitabine.

Previously [45], it was found that oxonium derivatives of *closo*-dodecaborate, such as compound **4** (Scheme 1), are able to react with various amines in high yields. In article [46], a selective alkylation of tertiary amines in the presence of hydroxyl groups using dioxonium derivative **4** was demonstrated. We used this approach to incorporate *closo*-dodecaborate moiety into gemcitabine’s core. For this purpose, we first benzoylated exocyclic amino group of gemcitabine similarly to the benzoylation of cytidine in [47]. Then, benzoyl derivative **2** (Scheme 1) was subjected to the transamination reaction according to the procedure outlined in [48]. Isolated compound **3** (Scheme 1) containing a tertiary amino group was alkylated using dioxonium derivative of *closo*-dodecaborate **4**, similarly to the published procedure in [46]. We slightly modified the protocol and used DMF instead of acetonitrile as a solvent in the reaction for better solubility of the reagents. Thus, compound **5** (Scheme 1), a conjugate of gemcitabine and *closo-*dodecaborate, was isolated. The structures of conjugate **5** and its precursors were confirmed by ^1^H, ^13^C, ^19^F NMR and ESI mass spectrometry.

In a separate experiment, we found that the reaction of 2′-deoxycytidine 5′-monophosphate with compound **4** readily proceeded with the formation of more lipophilic products with the same UV characteristics. On the other hand, 2-deoxycytidine did not react under the same conditions. It is most likely that phosphate group is the alkylation target in 2′-deoxycytidine 5′-monophosphate. Thus, it was important to carry out phosphorylation after the introduction of *closo-*dodecaborate to avoid side processes affecting the phosphate group during alkylation. Compound **5** was selectively 5′-monophosphated to give **6** (Scheme 1) using the protocol from the articles [49,50,51]. Conjugate **6** was isolated as its tris(triethylammonium) salt and characterized by NMR ^1^H, ^13^C, ^19^F and ESI mass.

**Figure 2:**
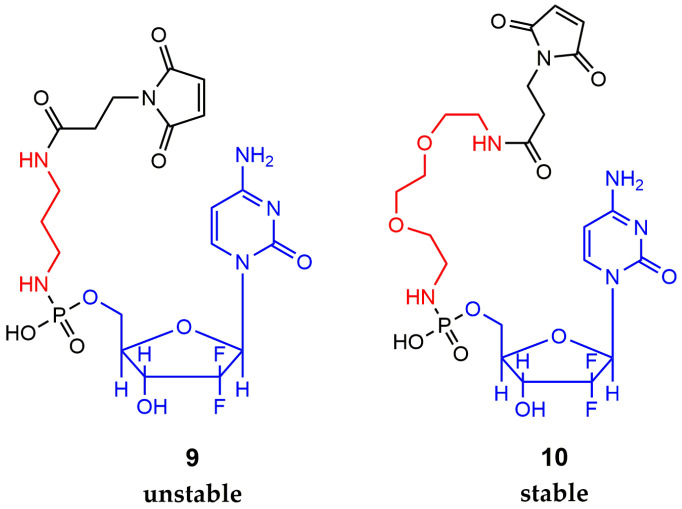
The structures of compounds **9** and **10**.

We then modified the phosphate group in **6** with a diamino linker. During the experiments, we found that the length of the linker between maleimide residue and phosphate is an important point for the stability of such conjugates. Compound **9** (Figure 2) containing a diaminopropyl linker was found to be unstable in water. We discovered the deficit of maleimide protons according to ^1^H NMR of compound **9** (see Supplementary Material). Further, in our attempts to purify **9**, it was found that this conjugate converted in several hours at RT into some product 9* (see Supplementary Material) with changed UV characteristics. NMR and mass spectra of this by-product support a hypothesis of hydrolysis of the maleimide core (see Supplementary Material) [52]. There are no maleimide protons in ^1^H NMR of 9*, and the main peak in its mass spectra corresponds to [M + H_2_O-1]^−^ of **9**. We proposed that a neighboring phosphate group could contribute to the hydrolysis process. Indeed, compound **10** (Figure 2) containing an extended 2,2′-(ethylenedioxy)bis(ethylamine) linker was found to be stable under the same conditions. So, we used this extended linker for the synthesis of the target compound **1**.

We relied on the published method in [53] to synthesize compound **7** (Scheme 1), but we changed the process of purification of the target product. The reaction of the maleimide derivative **8** and aliphatic amino group of **7** required completely removing the 2-thiopyridine and diamine impurities from **7** to avoid side reactions. We performed three-step purification and isolated the triethylammonium salt of the amine **7**. At the last step, activated pentafluorophenyl ether **8** was used for modification of the amine **7** according to the published method in [54]. Maleimide-containing derivative **1** was isolated as its bis(triethylammonium) salt with a high yield. ^1^H, ^13^C, ^19^F NMR and mass spectrometry data confirmed the structure of the target compound.

### 2.2. Bioconjugation

**Figure 3:**
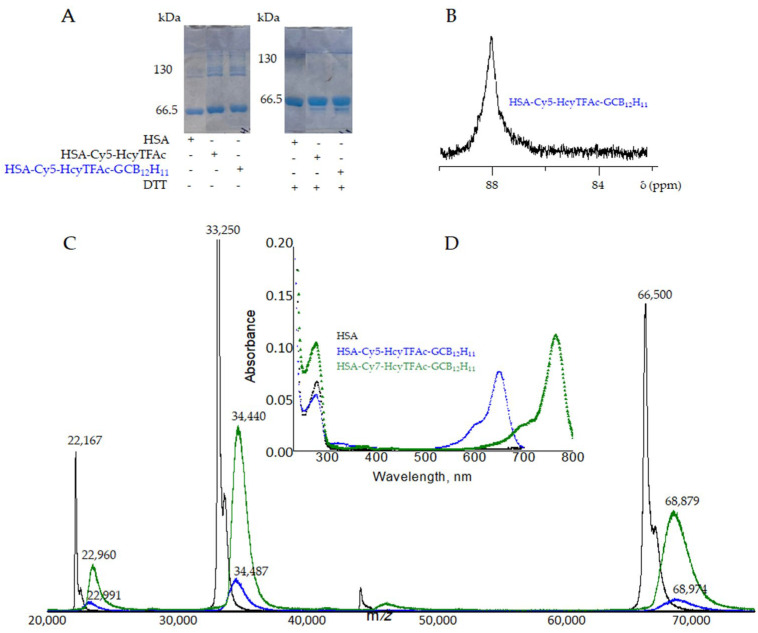
Characterizations of multifunctional human serum albumin conjugates. (**A**): SDS-PAGE of homocystamide conjugates of the HSA under Laemmli conditions with subsequent Coomassie blue staining. (**B**): ^19^F NMR spectrum (at 282.4 MHz) of HSA-Cy5-HcyTFAc-GCB_12_H_11_ (0.6 mM) in PBS buffer (pH 7.4; to provide deuterium lock, D_2_O was added to 20% of the total volume) at 37 °C. The chemical shifts are referred to the resonance of C_6_F_6_ at 0.0 ppm. (**C**): MALDI-TOF spectra of HSA (black line), HSA-Cy5-HcyTFAc-GCB_12_H_11_ (blue line) and HSA-Cy7-HcyTFAc-GCB_12_H_11_ (green line). (**D**): UV–vis spectra of HSA and its homocystamides in PBS buffer, pH 7.4. HSA—black; HSA-Cy5-HcyTFAc-GCB_12_H_11—_blue, HSA-Cy5-HcyTFAc-GCB_12_H_11—_green.

Earlier, our group synthesized fluorophore-labeled homocystamide conjugates of human serum albumin HSA-Cy5-HcyTFAc [30,32]. One-and-a-half copies of trifluoroacetate and a single copy of a fluorophore (Cy5 or Cy7) were covalently attached to a protein via suitable amino acids. This was confirmed by the electron spectroscopy data (Figure 3, panel D) and by MALDI-TOF mass spectroscopy (see Section 3.2.7. in ‘Methods’. The attachment of *N*-trifluoroacetylhomocysteine (HcyTFAc) was also confirmed by the presence of a signal at 88 ppm in the ^19^F NMR spectrum for all HSA conjugates (Figure 3, panel B; data for the conjugates with Cy7 are similar and not shown here).

**Scheme 2:**
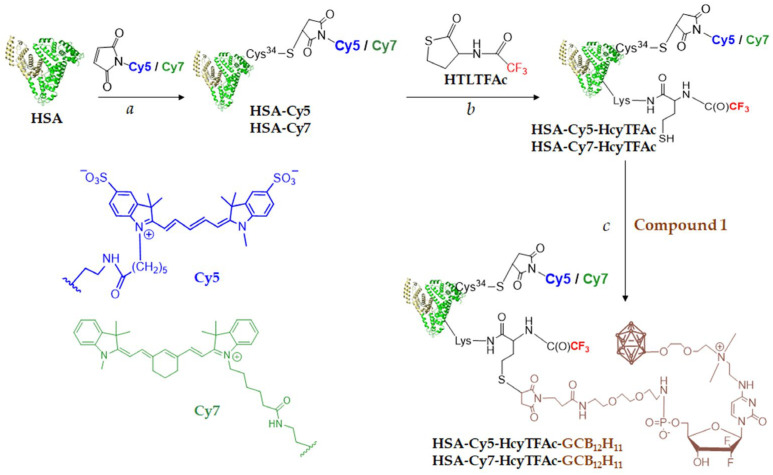
Synthetic route to obtain the multifunctionalized serum albumin conjugate—HSA—Cy5—HcyTFAc—GCB_12_H_11_ or HSA—Cy7—HcyTFAc—GCB_12_H_11_. Drug carrier (shown schematically as a heart-like structure)—human serum albumin (HSA); therapeutic agent, including in its structure the residues of gemcitabine and closo-dodecaborate–GCB_12_H_11_ (brown). Note that trifluoroacetyl homocycteine thiolactone (HTLTFAc) is used as a functional handle and a source of fluorine atoms (red). Optical imaging—fluorescent dye Cy5 (blue) or Cy7 (green) conjugated with Cys-34.

For the synthesis of the albumin-based theranostic agents HSA-Cy5-HcyTFAc-GCB_12_H_11_ and HSA-Cy7-HcyTFAc-GCB_12_H_11_, we used the reactivity of thiolactone (a cyclic thioester) as a latent thiol functionality in thiol-‘click’ chemistry. The thiol was released by nucleophilic ring opening (aminolysis) in amino groups on the HSA and, subsequently, reacted with a thiol ‘scavenger’ (a maleimide derivative of the gemcitabine, compound **1** (Scheme 2, path c). Modification of HSA results in the accumulation of oligomeric forms of the protein, which are unstable to the action of dithiothreite (Figure 3, panel A; data for the conjugates with Cy7 are the same as for Cy5 and are not shown here).

Modification of HSA-Cy5-HcyTFAc or HSA-Cy7-HcyTFAc with a threefold excess of the gemcitabine analogue (twofold excess relative to the number of mercapto groups on the conjugate) resulted in the addition of 1.5 residues of the gemcitabine analogue per protein molecule. This was confirmed by MALDI-TOF mass spectroscopy data (Figure 3C). The mass of HSA differs from the measured mass of HSA-Cy5-HcyTFAc-GCB_12_H_11_ by 2474 Da, which corresponds to 1.5 residues of the gemcitabine analogue. The same result was received for HSA-Cy7-HcyTFAc-GCB_12_H_11_; the mass difference was 2379 Da. Furthermore, it was confirmed by inductively coupled plasma atomic emission spectroscopy. The amount of boron in the sample HSA-Cy5-HcyTFAc-GCB_12_H_11_ was 18 times greater than the amount of albumin in it. The amount of boron in the sample HSA-Cy7-HcyTFAc-GCB_12_H_11_ was 18.3 times greater than the amount of albumin molecules. Thus, it corresponds to the 1.5 residues of *closo*-dodecarborate addition per protein molecule.

### 2.3. Cell Viability Assay

**Figure 4:**
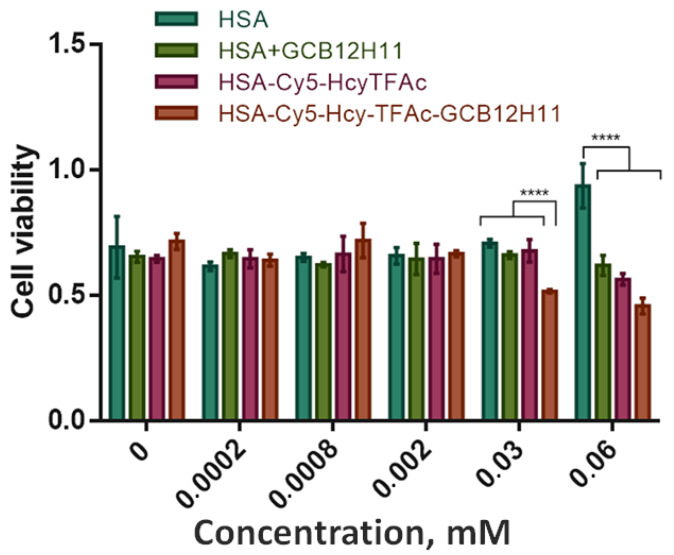
Viability of T98G cells. The cancer cell line was incubated for 24 h with HSA a mixture of HSA + gemcitabine, HSA-Cy5-HcyTFAc and HSA-Cy5-Hcy-TFAc-GCB_12_H_11_ at various concentrations. After incubation, the cell viability was measured using the MTT test. The reported values represent the mean ± SD (n = 3). The concentrations used were taken for the HSA component. The concentration of gemcitabine and its analogue in HSA + gemcitabine and HSA-Cy5-Hcy-TFAc-GCB_12_H_11_ was 1.5 parts of the HSA concentration. **** — *p*-value ≤ 0.0001.

The effect of the HSA-Cy5-Hcy-TFAc-GCB_12_H_11_ construct on viability of the glioblastoma T98G cell line was determined by standard MTT colorimetric assay [55]. The results are shown in Figure 4. In general, there were no significant differences in the viability of glioblastoma cells incubated with different albumin conjugates; however, the viability of T98G cells decreased in the presence of 0.03 mM or more HSA Cy5-Hcy-TFAc-GCB_12_H_11_ (*p*-value ≤ 0.0001).

The increase in cell survival in the presence of HSA (*p*-value ≤ 0.0001) can be explained by the stimulation of their growth by albumin as a food resource.

Cell survival in the presence of a mixture of HSA and gemcitabine did not decrease within the range of the used gemcitabine concentrations. In the presence of the HSA-Cy5-Hcy-TFAc-GCB_12_H_11_ conjugate, the same concentrations relative to gemcitabine resulted in a decrease in the number of surviving cells. The half-maximal inhibitory concentration (IC_50_) for HSA-Cy5-Hcy-TFAc-GCB_12_H_11_ was 0.47 mM with the correlation coefficient R = 0.82. This can be explained by the fact that gemcitabine penetrates into cancer cells better as part of the conjugate than as part of the noncovalent complex with albumin. Another explanation could be the greater efficacy of the gemcitabine analogue compared to gemcitabine itself. In any case, the covalent binding of the gemcitabine analogue to the albumin construct enhances its effectiveness.

Without neutron irradiation, the cell line retained a proliferation rate of over 70% upon treatment with the gemcitabine-containing boronated conjugate HSA-Cy5-Hcy-TFAc-GCB_12_H_11_ within its concentration range of 0.03–0.06 mM (Figure 4). Thus, a conjugate concentration of about 0.03 mM can be used to evaluate the effect of the drug on glioma cell colony formation in neutron irradiation experiments.

### 2.4. The Usage of the HSA-Cy7-HcyTFAc-GCB_12_H_11_ Conjugate as Boron Delivery Agent in BNCT

We performed the BNCT experiment using the HSA-Cy7-HcyTFAc-GCB_12_H_11_ conjugate as a boron delivery agent and ^10^B-boronophenylalanine as a positive control for BNCT. We used a clonogenic assay for evaluation of the BNCT effectiveness. The treatment of cells with the boron-modified conjugate and subsequent irradiation was carried out in the same way as in the previous work [30]. Irradiation of cell cultures was carried out at the BINP neutron source [56]. 

**Figure 5:**
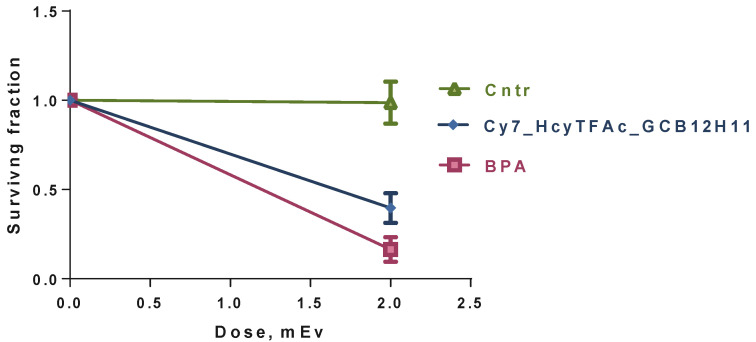
Surviving fractions of neutron-irradiated human glioblastoma cells T98G depending on the incubation of HSA-Cy7-HcyTFAc-GCB12H11 conjugate or BPA.

The survival fraction of T98G human glioblastoma cells upon incubation with the conjugate and subsequent irradiation with epithermal neutrons was ≈0.4 (Figure 5), which differed significantly from the control group (*p* ≤ 0.0001). Thus, the conjugate HSA-Cy7-HcyTFAc-GCB_12_H_11_ shows a synergistic effect between gemcitabine (it has a toxicity to cancer cells without irradiation) and *closo*-dodecaborate (toxicity to cancer cells is enhanced by neutron irradiation). 

In our previous work [30], we described a boron-containing albumin-based conjugate containing thionyl trifluoroacetate (TTFA). TTFA was attached to the protein at arginine residues, followed by treatment with boric acid. The conjugate with TTFA had a similar efficiency under irradiation with epithermal neutrons (*p* ≤ 0.0001) but at a higher neutron flux (4 × 10^12^ cm^−2^ for the conjugate with TTFA vs. 2.2 × 10^12^ cm^−2^ for the conjugate with gemcitabine).

It is known that TTFA binds to the active site in complex II of the electron transport chain and prevents the reduction in ubiquinone. That inhibits the electron transport chain and can be used to prevent an increase in hyperglycemia-induced mitochondrial reactive oxygen species [57]. Gemcitabine triphosphate has other targets. It competes with deoxycytidine triphosphate in DNA synthesis [58] and inhibits enzyme ribonucleotide reductase, which reduces ribonucleotides to deoxyribonucleotides. The resulting deficiency of cytidine-5’-triphosphate leads to increased incorporation of the triphosphate form of gemcitabine into DNA [59,60]. Different targets of the chemotherapeutic parts of the conjugates can cause a different synergistic effect on any type of cancer cell. It depends on the metabolism of the cancer tissue. Thus, combining a variety of chemotherapeutic agents with boron-containing molecules in a covalent conjugate is promising.

At the same time, the colonogenic assay revealed a decrease in viability with the use of ^10^B-boronophenylalanine containing the boron isotope–10 with the lowest surviving fraction compared to the HSA-Cy7-HcyTFAc-GCB_12_H_11_ conjugate (*p* ≤ 0.01). It is worth noting that, here, we used a HSA-Cy7-HcyTFAc-GCB_12_H_11_ conjugate made of natural boron which contains 20% ^10^B isotope. The BPA control contains solely ^10^B. Taking into account the number of boron atoms in one residue and the number of *closo*-dodecaborate residues attached to albumin, the amount of ^10^B in the control is five times greater than in the conjugate. On the one hand, the use of a conjugate containing pure ^10^B will lead to greater therapeutic efficiency. On the other hand, since the isolation of pure ^10^B isotope is a rather expensive technology, the use of natural boron for BNCT can simplify and reduce the cost of obtaining boron-containing compounds.

## 3. Materials and Methods

### 3.1. Materials

Human serum albumin (HSA) was obtained from Sigma–Aldrich Chem. Co. (St. Louis, MO, USA). The product number of HSA used was A3782. The concentrations of albumin solutions were determined by absorption at 278 nm, pH 7.4, using the molar extinction coefficient ***ε*** = 3.7 × 10^4^ M**^−^**^1^cm**^−^**^1^ [61].

The human glioblastoma T98G cell line was received from the Russian cell culture collection (Russian Branch of the ETCS, St. Petersburg, Russia).

1,4-Dioxonium derivative of *closo*-dodecaborate **4** was prepared according to the described method in [62]. Maleimide derivative **8** was kindly provided by Dr. L. S. Koroleva (ICBFM SB RAS, Novosibirsk, Russia).

Reagents and materials were purchased from Sigma-Aldrich (USA) and Reachem (Moscow, Russia), unless otherwise indicated. Milli-Q water with a conductivity greater than 18 MΩ/cm was used in all experiments. Phosphate buffered saline (PBS) (0.01 M, pH 7.3–7.5, Biolot). Organic solvents were dried and purified using standard procedures.

### 3.2. Methods

Monitoring of reaction progression (except bioconjugation) was performed on a Milichrom A02 chromatograph system equipped with the MultiChrom program package (Econova, Novosibirsk, Russia) on a ProntoSIL 125 C18 column (2 × 75 mm) in a gradient of buffer B (0.1 M triethylammonium acetate (TEAAc), pH 7.0, 80% MeCN) in buffer A (0.1 M TEAAc, pH 7.0, water) with an elution rate of 0.2 mL/min and UV detection at 250, 260, 280 and 300 nm. TLC was carried out on Kieselgel 60 F254 plates (Merck, Darmstadt, Germany) in the proper solvent systems and visualized by UV irradiation, ninhydrin (amine groups) or a saturated solution of palladium II chloride in 1 M hydrochloric acid (*closo*-dodecaborate anion). Preparative silica gel column chromatography was performed using silica gel 60 (40–63 μm/230–400 mesh) (Macherey-Nagel, Düren, Germany). Preparative silica gel column chromatography, reversed-phase chromatography (RPC) and ion exchange chromatography were performed using silica gel 60 (40–63 μm/230–400 mesh) (Macherey-Nagel, Germany), Porasil C 18 (55–105 μm, 125 A) (Waters, Milford, MA, USA), Servacel P23 (Serva, Heidelberg, Germany) and Q Sepharose Fast Flow (GE Healthcare, Chicago, IL, USA). All evaporations were performed under reduced pressure.

Electronic absorption spectra were acquired on a UV-1800 spectrometer (Shimadzu, Kyoto, Japan).

NMR spectra were acquired on Bruker AV-400, AV-300 instruments and DRX-500 (Bruker, Bremen, Germany) in appropriate deuterated solvents at 30 °C. Chemical shifts (δ) are reported in ppm relative to the tetramethylsilane (TMS), C_6_F_6_, 85% H_3_PO_4_ or D_2_O (*δ* 4.8 ppm) signals. Coupling constants *J* are reported in Hertz. 

Mass spectra of proteins and peptides were recorded on a Bruker Autoflex Speed (Bruker Daltonics, Germany) MALDI-TOF mass spectrometer in positive linear mode. A smartbeam-II laser was used. 2,5-Dihydroxyacetophenone (2,5-DHAP) was used as a matrix. Protein samples were desalted by ZipTip C4 pipette tips. An amount of 2 µL of the protein sample solution was mixed with 2 µL of a 2% TFA (trifluoroacetic acid). To the latter solution, 2 µL of the matrix (2,5-DHAP) was added. The mixture was pipetted up and down until crystallization started. Mass spectra were obtained by averaging 3000 laser shots. External calibration was provided by [M + H]^+^ HSA at *m/z* 66.5 kDa.

ESI mass spectra were registered on Agilent ESI MSD XCT Ion Trap (Agilent Technologies, Santa Clara, CA, USA) in positive or negative mode at The Joint Center for genomic, proteomic and metabolomics studies (IChBFM SB RAN, Novosibirsk, Russia). 

The boron content in the resulted protein conjugates was determined by inductively coupled plasma atomic emission spectrometry (ICP AES) on an ICPE-9820 high-resolution spectrometer (Shimadzu, Kyoto, Japan). The samples were not subjected to preliminary incineration and were diluted with deionized water to 7 mL. Calibration dependencies were built using a single-element standard solution Boron Standard (Sigma Aldrich) in the range of 0.01–10 mg/L. The result of the analysis was obtained by averaging over four analytical lines.

SDS-PAGE of human serum albumin conjugates were analyzed by sodium dodecyl sulfate polyacrylamide gel electrophoresis using 7% PAAG under Laemmli conditions without the addition of DTT or in the presence of DTT with subsequent Coomassie Brilliant Blue (BioRad) staining.

Low molecular weight materials (MW < 3 kDa) were removed from solutions of polymer conjugates by centrifugal filtration using Centricon concentrators with a MWCO of 3-kDa (Amicon Centriprep YM30, Millipore, Bedford, MA, USA).

#### 3.2.1. 4-*N*-Benzoyl-2′-deoxy-2′,2′-Difluorocytidine **2**

Gemcitabine (260 mg, 1 mmol) and benzoic anhydride (230 mg, 1 mmol) were dissolved in DMF (5 mL) and stirred at RT for 24 h. The reaction mixture was evaporated. Diethyl ether (10 mL) was added to the residue and the precipitate was filtered off. Yield: 294 mg, 0.80 mmol, 80%. ^1^H (DMSO-*d_6_*): 11.38 (1H, br s, NH), 8.32 (1H, d, *J* 7.5, H6), 8.01 (2H, d, *J* 7.7, *o*-Ph), 7.64 (1H, t, *J* 7.3, *p*-Ph), 7.52 (2H, t, *J* 7.6, *m*-Ph), 7.40 (1H, br d, *J* 7.5, H5), 6.34 (1H, br s, 3′-OH), 6.21 (1H, t, *J* 7.2, H1′), 5.34 (1H, br s, 5′-OH), 4.27–4.18 (1H, m, H3′), 3.94–3.89 (1H, m, H5′), 3.85–3.80 (1H, m, H5′), 3.71–3.65 (1H, m, H4′). ^13^C (DMSO-*d_6_*): 167.48, 163.63, 154.11, 144.63, 133.0, 132.82, 128.50, 128.42, 122.95 (t, *J_CF_ 259*), 96.67, 84.17 (t, *J_CF_ 31*), 81.04 (t, *J_CF_ 4*), 68.36 (t, *J_CF_ 22*), 58.77. ^19^F (DMSO-*d_6_*) 45.77 (br s, CF_2_).

#### 3.2.2. 4-*N*-(2-Dimethylaminoethyl)-2′deoxy-2′,2′-Difluorocytidine **3**

Triazabicyclodecene (TBD) (340 mg, 2.4 mmol) and *N,N*-dimethylethylenediamine (1.26 mL, 11 mmol) were added to a solution of **2** (294 mg, 0.8 mmol) in DMF (3 mL). The mixture was stirred at 60 °C for 2 h. After completion of the reaction, the mixture was evaporated, and the residue was washed with diethyl ether (2 × 20 mL). The crude product was purified by RPC in a linear gradient of EtOH in water (0–20%). Appropriate fractions were evaporated and passed through a column packed with Servacel P23 in H^+^ form. Elution was performed using a linear gradient of NH_4_HCO_3_ (0–0.3 M) in 20% aq. EtOH as an eluent. Fractions containing the product were combined and evaporated. Yield: 88 mg, 0.26 mmol, 33%. ^1^H NMR (D_2_O): 7.57 (1H, d, *J* 7.7, H6), 6.13 (1H, t, *J* 7.8, H1′), 5.92 (1H, d, *J* 7.7, H5), 4.31–4.21 (1H, m, H3′), 4.01–3.90 (2H, m, H5′), 3.82–3.75 (1H, m, H4′), 3.47 (2H, t, *J* 6.1, CH_2_), 2.60 (2H, t, *J* 6.1, CH_2_), 2.23 (3H, s, N(CH_3_)_2_). ^13^C NMR (D_2_O): 163.93, 157.22, 139.21, 121.99 (t, *J_CF_ 259*), 97.23, 84.40 (t, *J_CF_ 32*), 79.72 (t, *J_CF_ 4*), 69.00 (t, *J_CF_ 23*), 59.02, 56.22, 43.56, 37.47. ^19^F NMR (D_2_O): 45.85–45.67 (m, CF_2_). MS ESI (*m/z*): [M + H]^+^ calcd. for C_13_H_21_F_2_N_4_O_4_^+^ 335.15; found 334.90.

#### 3.2.3. Compound **5**

The mixture of **3** (85 mg, 0.25 mmol) and (Bu_4_N)^+^[B_12_H_11_O(CH_2_CH_2_)_2_O]^−^ (165 mg, 0.23 mmol) in DMF (2.6 mL) was stirred at 70 °C overnight. Then, the solvent was evaporated and the residue was purified by RPC using a linear gradient of EtOH in water (0–20%) as an eluent. Yield: 130 mg, 0.23 mmol, 92%. ^1^H NMR (D_2_O): 7.66 (1H, d, *J* 7.7, H6), 6.14 (1H, t, *J* 7.4, H1′), 6.05 (1H, d, *J* 7.7, H5), 4.32–4.23 (1H, m, H3′), 4.00–3.78 (7H, m, OCH_2_, NHCH_2_, H5′, H4′), 3.66–3.62 (2H, m, OCH_2_), 3.61–3.56 (6H, m, OCH_2_, CH_2_N^+^(CH_3_)_2_CH_2_), 3.18 (6H, br s, N^+^(CH_3_)_2_), 1.60–0.45 (11H, m, BH). ^13^C NMR (D_2_O): 164.03, 157.17, 139.86, 122.01 (t, *J_CF_ 260*), 97.37, 84.59 (t, *J_CF_ 31*), 79.77, 71.11, 68.89 (t, *J_CF_ 23*), 67.32, 63.57, 61.71, 59.07, 52.07, 44.82, 43.27, 36.38. ^19^F NMR (D_2_O): 45.69–45.30 (m, CF_2_). MS ESI (*m/z*): [M]^—^ calcd. for C_17_H_39_B_12_F_2_N_4_O_6_^—^ 563.40; found 563.20.

#### 3.2.4. Compound **6**

The mixture of **5** (90 mg, 0.16 mmol) and 1,8-bis(dimethylamino) naphthalene (40 mg, 0.19 mmol) was dissolved in trimethyl phosphate (1.3 mL). The mixture was cooled to 0 °C and phosphoryl chloride (0.023 mL, 0.24 mmol) was added. The reaction mixture was stirred for 1.5 h at RT. The reaction was terminated by adding 1M triethylammonium bicarbonate, pH 7.5, (TEAB) (10 mL). The mixture was stirred for 1 h and then evaporated. The residue was purified by ion exchange chromatography (Q Sepharose) using linear gradient of TEAB (0–1.0 M) in 20% aq. EtOH. Appropriate fractions were combined and evaporated. Yield: 91 mg, 0.096 mmol, 60%. ^1^H NMR (D_2_O):7,75 (1H, d, *J* 7.6, H6), 6.17 (1H, t, *J* 7.0, H1′), 6.06 (1H, d, *J* 7.6, H5), 4.42–4.33 (1H, m, H3′), 4.25–4.03 (3H, m, H5′, H4′), 3.91 (2H, br s, OCH_2_), 3.87 (2H, br t, *J* 6.2, CH_2_NH), 3.63 (2H, br t, *J* 6.5, N^+^CH_2_), 3.61–3.57 (6H, m, OCH_2_, OCH_2_, N^+^CH_2_), 3.19–3.16 (6H, s, N^+^(CH_3_)_2_), 1.74–0.56 (11H, m, BH). ^13^C NMR (D_2_O): 164.09, 157.09, 139.85, 121.96 (t, *J_CF_ 258*), 97.36, 84.42 (t, *J_CF_ 32*), 78.55, 71.13, 68.34 (t, *J_CF_ 23*), 67.30, 63.58, 62.02, 58.24, 51.95, 46.37, 42.02, 34.24, 7.98. ^19^F NMR (D_2_O): 45.30 (br s, CF_2_). ^31^P NMR (D_2_O): 0.17 (s). MS ESI (*m/z*): [M]^—^ calcd. for C_17_H_40_B_12_F_2_N_4_O_9_P^−^ 643.37; found 643.00.

#### 3.2.5. Compound **7**

The mixture of **6** (70 mg, 0.07 mmol), triphenylphosphine (104 mg, 0.4 mmol), 2,2’-dipyridyl disulfide (88 mg, 0.4 mmol) and *N*-methylimidazole (0.28 mL, 4 mmol) in DMSO (1 mL) was stirred for 30 min at RT. Then, 1,2-bis(2-aminoethoxy)ethane (0.15 mL, 1 mmol) was added. The reaction mixture was stirred for 1 h at RT and then poured into the mixture of acetone/diethyl ester (1/1). The precipitate formed was filtered off and purified by RPC in a linear gradient of EtOH in water (0–20%). Appropriate fractions were evaporated and passed through a column packed with Servacel P23 in H^+^ form. Elution was performed using a linear gradient of NH_4_HCO_3_ (0–0.3 M) in 20% aq. EtOH as an eluent. Appropriate fractions were combined, evaporated and purified by anion exchange chromatography (Q Sepharose) using a linear gradient of TEAB (0–1.0 M) in 20% aq. EtOH as eluent. Fractions containing the product were combined and evaporated. Yield: 26 mg, 0.03 mmol, 43%. ^1^H NMR (D_2_O): 7.64 (1H, d, *J* 7.6, H6), 6.06 (1H, t, *J* 7.0, H1′), 5.98 (1H, d, *J* 7.6, H5), 4.31–4.23 (1H, m H3′), 4.05–3.98 (2H, m, H5′), 3.92–3.87 (1H, m, H4′), 3.80 (2H, br s, OCH_2_), 3.76 (2H, br t, *J* 5.8, NHCH_2_CH_2_N^+^), 3.60–3.56 (2H, br t, *J* 5.8, NHCH_2_CH_2_N^+^), 3.54–3.50 (6H, m, OCH_2_CH_2_O, OCH_2_), 3.49–3.45 (6H, m, OCH_2_CH_2_O, OCH_2_), 3.41 (2H, t, *J* 5.6, N^+^CH_2_), 3.34–3.26 (4H, m, CH_2_NHP, CH_2_NH_2_), 3.07 (6H, s, N^+^(CH_3_)_2_), 1.63–0.40 (11H, m, BH). ^13^C NMR (D_2_O): 164.12, 157.15, 139.85, 122.07 (t, *J_CF_ 259*), 97.50, 84.45 (t, *J_CF_ 31*), 78.66, 71.19, 70.95, 69.20, 68.47 (t, *J_CF_ 23*), 67.37, 66.12, 63.60, 61.75, 61.47, 58.39, 52.10, 46.41, 42.03, 40.32, 38.74, 34.25, 8.01. ^19^F NMR (D_2_O): 45.69–45.30 (m, CF_2_). ^31^P NMR (D_2_O): 9.31 (s). MS ESI (*m/z*): [M]^−^ calcd. for C_23_H_54_B_12_F_2_N_6_O_10_P^—^ 773.48; found 773.10; [M − H]^2—^calcd. for C_30_H_53_B_12_F_2_N_6_O_10_P^2—^ 386.24; found 385.90.

#### 3.2.6. Compound **1**

The solution of maleimide derivative **8** (11 mg, 0.033 mmol) in DMF (0.1 mL) was added to a mixture of **6** (26 mg, 0.03 mmol) and TEA (0.02 mL, 0.14 mmol) in DMF (1.0 mL). The reaction mixture was stirred for 30 min at RT and then poured in diethyl ester (8 mL). The precipitate formed was filtered off and dissolved in water (0.4 mL). The acetone/diethyl ester mixture (1/1) was added to the solution and the mixture was chilled at −20 °C. The precipitate formed was separated by centrifugation, washed with diethyl ether and dried. Yield: 0.037 mg, 0.029 mmol, 97%. ^1^H NMR (D_2_O): 7.65 (1H, d, *J* 7.6, H6), 6.67 (2H, s, CH = CH), 6.06 (1H, t, *J* 6.9, H1′), 5.98 (1H, d, *J* 7.6, H5), 4.32–4.22 (1H, m, H3′), 4.05–3.98 (2H, m, H5′), 3.92–3.86 (1H, m, H4′), 3.83–3.78 (2H, m, OCH_2_), 3.76 (2H, br t, *J* 6.4, NHCH_2_CH_2_N^+^), 3.59 (2H, t, *J* 6.1, CH_2_CO), 3.52 (2H, br t, *J* 6.8, NHCH_2_CH_2_N^+^), 3.51–3.43 (12H, m, OCH_2_CH_2_O, OCH_2_CH_2_O, OCH_2_, N^+^CH_2_), 3.42–3.34 (6H, m, OCH_2_, CH_2_NH_2_, CH_2_NHP), 3.12 (2H, br t, *J* 5.1, CH_2_N), 3.09–3.06 (6H, s, N^+^(CH_3_)_2_), 1.64–0.43 (11H, m, BH). ^13^C NMR (D_2_O): 173.31, 172.26, 164.11, 157.13, 139.87, 134.20, 122.06 (t, *J_CF_ 258*), 97.46, 84.45 (t, *J_CF_ 32*), 78.66, 71.18, 70.96, 69.14, 68.45 (t, *J* 22), 68.40, 67.35, 65.78, 63.62, 61.85, 61.38, 58.34, 52.05, 46.42, 40.31, 38.76, 34.49, 34.25, 29.99, 7.99. ^19^F NMR (D_2_O): 45.40 (br s, CF_2_). ^31^P NMR (D_2_O): 9.30 (s). MS ESI (*m/z*): [M]^—^ calcd. for C_30_H_59_B_12_F_2_N_7_O_13_P^—^ 924.51; found 924.20; [M − H]^2—^ calcd. for C_30_H_58_B_12_F_2_N_7_O_13_P^2—^ 461.75; found 461.40.

#### 3.2.7. Synthesis and Characterization of Multifunctional Human Serum Albumin-Therapeutic Conjugates HSA-Cy5-HcyTFAc-GCB_12_H_11_ and HSA-Cy7-HcyTFAc-GCB_12_H_11_

HSA-Cy5 and HSA-Cy7 were synthetized as described in [35].

HSA-Cy5-HcyTFAc and HSA-Cy7-HcyTFAc were synthetized as described in [30].

Purified HSA-Cy5-TFAc or HSA-Cy7-HcyTFAc in PBS buffer (767 µL, 0.7 × 10^−3^ M^−1^L^−1^) was mixed with compound 1 (2.72 mg, 45 µL and 2.4165 µmol) dissolved in DMSO, and it was ensured that the molar amount of B_12_H_11_ was 4.5 times that of has, and the volume of B_12_H_11_ was 0.058 times that of HSA. The reaction was carried out for 18 h at 37 °C. Purification of the final conjugate was carried out using centricons (Amicon Centriprep YM30, Millipore, Bedford, MA) that pass molecules with a molecular weight of less than 3000 Da. For washing, 10% (by volume) DMSO in PBS (10 volumes of the reaction mixture), and then PBS (10 volumes of the reaction mixture) were used.

The yield of HSA-Cy5-HcyTFAc-GCB_12_H_11_ derivatives was ~64%. UV–vis (PBS buffer, pH 7.4): *λ*_max_ 278 nm (*ε* = (5.1 ± 0.1) × 10^4^), *λ*_max_ 646 nm (*ε* = (2.7 ± 0.1) × 10^5^). ^19^F NMR (PBS buffer, pH 7.4, D_2_O to 20% of the total volume) 88.0 (*N*-trifluoroacetyl residue). Mass spectrometry (MALDI-TOF) m/z: measured MW for HSA is 66,500 Da; calculated MW for HSA-Cy5-HcyTFAc is 67,480 Da (MW_HSA_ + MW_Cy5_ + MW_HcyTFAc_); measured MW for HSA-Cy5-HcyTFAc is 67,587 Da that corresponds to 1.5 HcyTFAc residue connected per HSA-Cy5 molecule; calculated MW for HSA-Cy5-HcyTFAc-GCB_12_H_11_ is 68,511 Da (MW_HSA_ + MW_Cy5_ + 1.5 MW_HcyTFAc_ + MW_GCB12H11_); measured MW for HSA-Cy5-HcyTFAc-GCB_12_H_11_ is 68,974 Da, that corresponds to 1.5 GCB_12_H_11_ residue connected per albumin molecule. Inductively coupled plasma atomic emission spectroscopy: 1.02 ppm of boron (total) was detected at a conjugate quantity in the analyzed sample 0.035 μmol (Figure S10, Supporting Information). It corresponded to 1.5 GCB_12_H_11_ residues per albumin.

The yield of HSA-Cy7-HcyTFAc-GCB_12_H_11_ derivatives was ~60%. UV–vis (PBS buffer, pH 7.4): *λ*_max_ 278 nm (*ε* = (7.2 ± 0.1) × 10^4^), *λ*_max_ 762 nm (*ε* = (1.9 ± 0.1) × 10^5^). ^19^F NMR (PBS buffer, pH 7.4, D_2_O to 20% of the total volume) 88.0 (*N*-trifluoroacetyl residue). Mass spectrometry (MALDI-TOF) m/z: measured MW for HSA is 66,500 Da; calculated MW for HSA-Cy7-HcyTFAc is 67,385 Da (MW_HSA_ + MW_Cy7_ + MW_HcyTFAc_); measured MW for HSA-Cy7-HcyTFAc is 67,493 Da that corresponds to 1.5 HcyTFAc residue connected per HSA-Cy7 molecule; calculated MW for HSA-Cy7-HcyTFAc-GCB_12_H_11_ is 68,416 Da (MW_HSA_ + MW_Cy7_ + 1.5 MW_HcyTFAc_ + MW_GCB12H11_); measured MW for HSA-Cy5-HcyTFAc-GCB_12_H_11_ is 68,974 Da, that corresponds to 1.5 GCB_12_H_11_ residue connected per albumin molecule. Inductively coupled plasma atomic emission spectroscopy: 0.296 ppm of boron (total) was detected at a conjugate quantity in the analyzed sample 0.01 μmol (Figure S10, Supporting Information). It corresponded to 1.5 GCB_12_H_11_ residues per albumin.

#### 3.2.8. Cell Viability Assay (MTT Test)

To determine the cytotoxicity of the conjugates, a T98G human glioblastoma cell line was used as well as the previously described protocol with minor modifications [55]. αMEM nutrient medium containing 10% by volume of fetal bovine serum and 1% solution of antimycotic/antibiotics was used. In the wells of a 96-well plate, 100 μL of cell suspension was added in the amount of 5 × 10^3^ cells per 1 well in the αMEM nutrient medium containing 1% solution of antimycotic/antibiotics.

The filled plate was incubated for 24 h at (37.0 ± 1.0) °C in an atmosphere of (5.0 ± 0.5)% CO_2_. After 24 h, six dilutions of the conjugates were prepared in the αMEM nutrient medium containing a 1% solution of antimycotic antibiotics with a final conjugate concentration in the well of 0.6, 0.3, 0.02, 0.002, 0.0008, 0.0002 and 0 mM. The obtained dilutions of the test conjugate were added (100 μL of each one) to the wells of the plate. The filled plate was incubated for 24 h at (37.0 ± 1.0) °C in an atmosphere of (5.0 ± 0.5)% CO_2_. After that, the medium was removed, and 200 μL of the appropriate nutrient medium containing 0.25 mg/mL MTT was added to each well. Incubation continued under the same conditions for 4 h. Then, the medium and MTT were removed from the wells, 100 μL of dimethyl sulfoxide was added and the optical density was measured on a multichannel flatbed scanner at a wavelength of λ = 570 nm, ref. 620 nm. The wells without protein samples were used as the zero level for the flatbed scanner.

Then, the mean absorbance and standard deviation were determined in the control wells and for each conjugate dilution concentration. Samples with the conjugate concentration 0 mg/mL were taken as control. Considering the obtained values of the optical density for each concentration of dilution of the conjugate and control samples, the percentage of surviving cells was calculated using Formula (1):

Cell viability = AAV_sample_./AAV_control_* (1)

* AAV_sample._—average absorption value in the wells with the sample; AAV_control_—average absorption value in the wells without the sample. 

The calculation of IC50 was carried out according to the protocol presented on the website https://www.sciencegateway.org/protocols/cellbio/drug/hcic50.htm (accessed on 14 October 2022).

Statistical analysis was performed in the following manner. Parametric data are expressed as mean ± standard deviation (SD). Each experiment was repeated at least three times. Statistical analysis was performed using GraphPad Prism 6.01 (GraphPad Software, San Diego, CA, USA). To compare more than two datasets, we used two-way ANOVA with multiple comparisons. Differences were considered significant if the *p* value was <0.05.

#### 3.2.9. Model BNCT Experiments

##### Incubation of the Cell Lines and Irradiation

Human glioblastoma T98G cells were incubated with HSA-Cy5-HcyTFAc-GCB_12_H_11_ conjugates (31 µM) then washed by 1 mL of complete culture medium. T98G cells incubated with ^10^B-4-borono-L-phenylalanine (50 ppm) for 18h were used as a positive control. Irradiated cells and non-irradiated cells not incubated with any boron compounds served as negative controls.

Neutron irradiation was performed at the accelerator-based epithermal neutron source at the Budker Institute of Nuclear Physics, Novosibirsk, Russia [56]. The layout of the facility is shown in Figure S11 (see Supporting Information).

The neutron source comprises an original design tandem accelerator (vacuum-insulated tandem accelerator VITA), a thin solid lithium target and a neutron beam shaping assembly. The facility can displace a lithium target in 5 positions; in Figure 1, they are marked as Positions A, B, C, D, E. In these studies, the lithium target was placed in Position *A*. The PMMA disk with a diameter of 200 mm and a thickness of 72 mm was placed below and close to the target. This disk served as a neutron moderator. The studied cell cultures in test tubes were placed inside a PMMA disk 200 mm in diameter and 40 mm thick at a distance of 25 mm from the center. This disk with test tubes was placed under the moderator with a gap of 10 mm. Cell cultures were irradiated at a proton beam energy of 2.1 MeV and a current of 1.35 mA for 92 min. The neutron flux density at the exit from the moderator was 4 10^8^ cm^−2^ s^−1^. Therefore, cell cultures were irradiated with a neutron fluence of 2.2 10^12^ cm^−2^.

##### Clonogenic Assay

Irradiated T98G glioblastoma cell lines were seeded in 6-well plates (TPP, Switzerland) at a density of 500 cells per well. The cells were incubated for a week at 37 °C in a humidified incubator under 5% (*v*/*v*) CO_2_. Clonogenic analysis was performed according to the method described earlier [63]. The calculation of the plating efficiency and surviving fraction was based on the survival of non-irradiated cells.

## 4. Conclusions

The synthesis of a gemcitabine analogue containing *closo*-dodecaborate can provide a therapeutic agent with a dual nature: it is both a chemotherapeutic agent as well as a tool for BNCT. The covalent attachment of a gemcitabine analogue to a macromolecular carrier such as HSA has multiple goals: increasing the lifetime of the drug in the bloodstream and the specificity of the therapeutic agent to tumor tissue. The albumin core allows the decoration of the final construct with dyes for its detection inside the body: fluorescent dyes (Cy5 or Cy7) or fluorine atoms for MRI. The HSA-Cy5-Hcy-TFAc-GCB_12_H_11_ conjugate has a noticeable toxicity against the human glioblastoma T98G cell line at protein concentrations of 0.03–0.06 mM. The half-maximal inhibitory concentration (IC_50_) for HSA-Cy5-Hcy-TFAc-GCB_12_H_11_ is 0.47 mM with correlation coefficient R = 0.82. Experiments with the use of the HSA-Cy7-Hcy-TFAc-GCB_12_H_11_ conjugate in BNCT show a decrease in the viability of tumor T98G cells under irradiation with epithermal neutrons with the neutron flux 2.2 × 10^12^ cm^−2^. Taking into account the toxicity of the conjugate against cancer cells without irradiation, the synergistic effect of gemcitabine and *closo-*dodecaborate residues takes place. Thus, a design of this type is promising for combination therapy.

## Data Availability

Not applicable.

## Supplementary Materials

The following supporting information can be downloaded at: https://www.mdpi.com/article/10.3390/molecules28062672/s1, Figure S1: Characterisics of 4-N-benzoyl-2′-deoxy-2′,2′-difluorocytidine.; Figure S2: Characterisics of 4-N-(2-dimethylaminoethyl)-2′-deoxy-2′,2′-difluorocytidine.; Figure S3: Characterisics of H+[Compound 5].; Figure S4: Characterisics of [Compound 6](TEA)3.; Figure S5: Characterisics of [Compound 7](TEA).; Figure S6: Characterisics of [Compound 1](TEA)2.; Figure S7: Characterisics of compound 9.; Figure S8: Characterisics of compound 9*.; Figure S9: Characterisics of compound 10.; Figure S10: Inductively coupled plasma atomic emission spectrometry (ICP AES) for HSA Cy5 HcyTFAc-GCB12H11 (a) and HSA Cy7 HcyTFAc-GCB12H11 (b). Boron spectral lines 182.640, 208.959, 249.678 and 249.773 nm.; Figure S11: Layout of the experimental facility: 1—vacuum-insulated tandem accelerator, 2—bending magnet, 3—lithium target, 4—beam shaping assembly. A, B, C, D, E—lithium target placement positions.

## Sample Availability

Samples of the following compounds are available from the authors: 4-*N*-Benzoyl-2′-deoxy-2′,2′-difluorocytidine, 4-*N*-(2-Dimethylaminoethyl)-2′deoxy-2′,2′-difluorocytidine, compounds **1** and **5**–**7**, conjugate HSA-Cy5-HcyTFAc-GCB_12_H_11_.

## References

[1] Paroha S., Verma J., Dubey R.D., Dewangan R.P., Molugulu N., Bapat R.A., Sahoo P.K., Kesharwani P. (2021). Recent advances and prospects in gemcitabine drug delivery systems. Int. J. Pharm..

[2] Dixon D.A., Smart B.E. (1991). Conformational energies of 2-fluoroethanol and 2- fluoroacetaldehyde enol—Strength of the internal hydrogen- bond. J. Phys. Chem..

[3] Mohammadian M., Kouchakzadeh H., Rahmandoust M., Mohammadian T. (2020). Targeted albumin nanoparticles for the enhancement of gemcitabine toxicity on cancerous cells. J. Drug Deliv. Sci. Technol..

[4] Bhushan B., Khanadeev V., Khlebtsov B., Khlebtsov N., Gopinath P. (2017). Impact of albumin based approaches in nanomedicine: Imaging, targeting and drug delivery. Adv. Colloid Interface Sci..

[5] Kulluru L.P., Rizvi S.A., D’Souza M., D’Souza M. (2013). Formulation development of albumin based theranostic nanoparticles as a potential delivery system for tumor targeting. J. Drug Target..

[6] Yhee J.Y., Lee J., Chang H., Jeewon L., Kwon I.C., Kim K. (2015). Molecular imaging and targeted drug delivery using albumin-based nanoparticles. Curr. Pharm. Des..

[7] Elzoghby A.O., Samy W.M., Elgindy S.N. (2012). Albumin-based nanoparticles as potential controlled release drug delivery systems. J. Control. Release.

[8] Bolaños K., Kogan M.J., Araya E. (2019). Capping gold nanoparticles with albumin to improve their biomedical properties. Int. J. Nanomed..

[9] Larsen M.T., Kuhlmann M., Hvam M.L., Howard K.A. (2016). Albumin-based drug delivery: Harnessing nature to cure disease. Mol. Cell. Ther..

[10] Schäffler M., Sousa F., Wenk A., Sitia L., Hirn S., Schleh C., Haberl N., Violatto M., Canovi M., Andreozzi P. (2014). Blood protein coating of gold nanoparticles as potential tool for organ targeting. Biomaterials.

[11] Brekken R.A., Sage E.H., Brekken R.A. (2001). Mini review SPARC, a matricellular protein: At the crossroads of cell matrix SPARC, a matricellular protein: At the crossroads of cell matrix communication. Matrix Biol..

[12] Kouros M. (1999). SPARC (osteonectin/BM-40). Int. J. Biochem. Cell Biol..

[13] Podhajcer O.L., Benedetti L.G., Girotti M.R., Prada F., Salvatierra E., Llera A.S. (2008). The role of the matricellular protein SPARC in the dynamic interaction between the tumor and the host. Cancer Metastasis Rev..

[14] Shi Q., Bao S., Song L., Wu Q., Bigner D.D., Hjelmeland A.B., Rich J.N. (2007). Targeting SPARC expression decreases glioma cellular survival and invasion associated with reduced activities of FAK and ILK kinases. Oncogene.

[15] Ali M.S., Al-Lohedan H.A. (2022). Experimental and Computational Investigation on the Interaction of Anticancer Drug Gemcitabine with Human Plasma Protein: Effect of Copresence of Ibuprofen on the Binding. Molecules.

[16] Ma T., Jiang J.L., Qi W.X., Chen J.Y., Xu H.P. (2022). A Novel Delivery System of RGD-HSA Loaded GEM/CUR Nanoparticles for the Treatment of Pancreatic Cancer Therapy. Drug. Des. Devel. Ther..

[17] Kong L., Du J., Gu J., Deng J., Guo Y., Tao B., Jin C., Fu D., Li J. (2022). Gemcitabine-Loaded Albumin Nanoparticle Exerts an Antitumor Effect on Gemcitabine-Resistant Pancreatic Cancer Cells Induced by MDR1 and MRP1 Overexpression in Vitro. Front. Surg..

[18] Yu X., Zhu W., Di Y., Gu J., Guo Z., Li H., Fu D., Jin C. (2017). Triple-functional albumin-based nanoparticles for combined chemotherapy and photodynamic therapy of pancreatic cancer with lymphatic metastases. Int. J. Nanomed..

[19] Guo Z., Wang F., Di Y., Yao L., Yu X., Fu D., Li J., Jin C. (2018). Antitumor effect of gemcitabine-loaded albumin nanoparticle on gemcitabine-resistant pancreatic cancer induced by low hENT1 expression. Int. J. Nanomed..

[20] Wang X., Liang Y., Fei S., He H., Zhang Y., Yin T., Tang X. (2018). Formulation and Pharmacokinetics of HSA-core and PLGA-shell Nanoparticles for Delivering Gemcitabine. AAPS Pharm. Sci. Tech..

[21] Norouzi P., Amini M., Mottaghitalab F., Mirzazadeh Tekie F.S., Dinarvand R., Mirzaie Z.H., Atyabi F. (2020). Design and fabrication of dual-targeted delivery system based on gemcitabine-conjugated human serum albumin nanoparticles. Chem. Biol. Drug. Des..

[22] Han H., Wang J., Chen T., Yin L., Jin Q., Ji J. (2017). Enzyme-sensitive gemcitabine conjugated albumin nanoparticles as a versatile theranostic nanoplatform for pancreatic cancer treatment. J. Colloid. Interface Sci..

[23] Bibi S., Ur-Rehman S., Khalid L., Bhatti I.A., Bhatti H.N., Iqbal J., Bai F.Q., Zhang H.X. (2022). Investigation of the adsorption properties of gemcitabine anticancer drug with metal-doped boron nitride fullerenes as a drug-delivery carrier: A DFT study. RSC Adv..

[24] Farzad F., Hashemzadeh H. (2020). Probing the effect of polyethene glycol on the adsorption mechanisms of Gem on the hexagonal boron nitride as a highly efficient polymer-based drug delivery system: DFT, classical MD and Well-tempered Metadynamics simulations. J. Mol. Graph. Model..

[25] Matsushita K., Okuda T., Mori S., Konno M., Eguchi H., Asai A., Koseki J., Iwagami Y., Yamada D., Akita H. (2019). A Hydrogen Peroxide Activatable Gemcitabine Prodrug for the Selective Treatment of Pancreatic Ductal Adenocarcinoma. Chem. Med. Chem..

[26] Xu Y., Huang Y., Lu W., Liu S., Xiao Y., Yu J. (2019). 4-Carboxyphenylboronic acid-decorated, redox-sensitive rod-shaped nano-micelles fabricated through co-assembling strategy for active targeting and synergistic co-delivery of camptothecin and gemcitabine. Eur. J. Pharm. Biopharm..

[27] Samaniego Lopez C., Martínez J.H., Acebedo S.L., Spagnuolo C.C. (2019). Benzoxaboroles as dynamic covalent receptors for bioconjugation and transport of nucleosides and related drugs: Proof of action in HeLa cells. Bioorg. Chem..

[28] Evens A.M., Rosen S.T., Helenowski I., Kline J., Larsen A., Colvin J., Winter J.N., van Besien K.M., Gordon L.I., Smith S.M. (2013). A phase I/II trial of bortezomib combined concurrently with gemcitabine for relapsed or refractory DLBCL and peripheral T-cell lymphomas. Br. J. Haematol..

[29] Lisitskiy V.A., Khan H., Popova T.V., Chubarov A.S., Zakharova O.D., Akulov A.E., Shevelev O.B., Zavjalov E.L., Koptyug I.V., Moshkin M.P. (2017). Multifunctional human serum albumin-therapeutic nucleotide conjugate with redox and pH-sensitive drug release mechanism for cancer theranostics. Bioorg. Med. Chem. Lett..

[30] Popova T.V., Dymova M.A., Koroleva L.S., Zakharova O.D., Lisitskiy V.A., Raskolupova V.I., Sycheva T.V., Taskaev S.Y., Silnikov V.N., Godovikova T.S. (2021). Homocystamide Conjugates of Human Serum Albumin as A Platform to Prepare Bimodal Multidrug Delivery Systems for Boron Neutron Capture Therapy. Molecules.

[31] Raskolupova V.I., Popova T.V., Zakharova O.D., Nikotina A.E., Abramova T.V., Silnikov V.N. (2021). Human Serum Albumin Labelling with a New BODIPY Dye Having a Large Stokes Shift. Molecules.

[32] Popova T.V., Pyshnaya I.A., Zakharova O.D., Akulov A.E., Shevelev O.B., Poletaeva J., Zavjalov E.L., Silnikov V.N., Ryabchikova E.I., Godovikova T.S. (2021). Rational design of albumin theranostic conjugates for gold nanoparticles anticancer drugs: Where the seed meets the soil?. Biomedicines.

[33] Popova T.V., Krumkacheva O.A., Burmakova A.S., Spitsyna A.S., Zakharova O.D., Lisitskiy V.A., Kirilyuk I.A., Silnikov V.N., Bowman M.K., Bagryanskaya E.G. (2020). Protein modification by thiolactone homocysteine chemistry: A multifunctionalized human serum albumin theranostic. RSC Med. Chem..

[34] Popova T.V., Khan H., Chubarov A.S., Lisitskiy V.A., Antonova N.M., Akulov A.E., Shevelev O.B., Zavjalov E.L., Silnikov V.N., Ahmad S. (2018). Biotin-decorated anti-cancer nucleotide theranostic conjugate of human serum albumin: Where the seed meets the soil?. Bioorg. Med. Chem. Lett..

[35] Chubarov A.S., Zakharova O.D., Koval O.A., Romaschenko A.V., Akulov A.E., Zavjalov E.L., Razumov I.A., Koptyug I.V., Knorre D.G., Godovikova T.S. (2015). Design of protein homocystamides with enhanced tumor uptake properties for 19F magnetic resonance imaging. Bioorg. Med. Chem..

[36] Moysan E., Bastiat G., Benoit J.P. (2013). Gemcitabine versus modified gemcitabine: A review of several promising chemical modifications. Mol. Pharm..

[37] Pereira M., Vale N. (2022). Two Possible Strategies for Drug Modification of Gemcitabine and Future Contributions to Personalized Medicine. Molecules.

[38] Dosio F., Brusa P., Crosasso P., Arpicco S., Cattel L. (1997). Preparation, characterization and properties in vitro and in vivo of a paclitaxel–albumin conjugate. J. Control. Release.

[39] Duncan R., Kopecková-Rejmanová P., Strohalm J., Hume I., Cable H.C., Pohl J., Lloyd J.B., Kopecek J. (1987). Anticancer agents coupled to N-(2-hydroxypropyl) methacrylamide copolymers. I. Evaluation of daunomycin and puromycin conjugates in vitro. Br. J. Cancer.

[40] Immordino M.L., Brusa P., Rocco F., Arpicco S., Ceruti M., Cattel L. (2004). Preparation, characterization, cytotoxicity and pharmacokinetics of liposomes containing lipophilic gemcitabine prodrugs. J. Control. Release.

[41] Plunkett W., Huang P., Gandhi V. (1995). Preclinical characteristics of gemcitabine. Anticancer Drugs.

[42] Huang P., Chubb S., Hertel L.W., Grindey G.B., Plunkett W. (1991). Action of 2’2’-Difluorodeoxycytidine on DNA Synthesis. Cancer Res..

[43] Peters G.J., van der Wilt C.L., van Moorsel C.J., Kroep J.R., Bergman A.M., Ackland S.P. (2000). Basis for effective combination cancer chemotherapy with antimetabolites. Pharm. Ther..

[44] Mini E., Nobili S., Casiagli B., Landini I., Mazzei T. (2006). Cellular pharmacology of gemcitabine. Ann. Oncol..

[45] Semioshkin A., Nizhnik E., Godovikov I., Starikova Z., Bregadze V. (2007). Reactions of oxonium derivatives of [B_12_H_12_]^2−^ with amines: Synthesis and structure of novel B_12_-based ammonium salts and amino acids. J. Organomet. Chem..

[46] Semioshkin A., Laskova J., Zhidkova O., Godovikov I., Starikova Z., Bregadze V., Gabel D. (2010). Synthesis and structure of novel *closo*-dodecaborate-based glycerols. J. Organomet. Chem..

[47] Bhat V., Ugarkar B.G., Sayeed V.A., Grimm K., Kosora N., Domenico P.A., Stocker E. (1989). A Simple and Convenient Method for the Selective *N*-Acylations of Cytosine. Nucleos. Nucleot. Nnucl..

[48] Abramova T.V., Vasil’eva S.V., Ivanova T.M., Shishkin G.V., Sil’nikov V.N. (2004). Monomers for Oligonucleotide Synthesis with Linkers Carrying Reactive Residues: I. The Synthesis of Deoxynucleoside Derivatives with Methoxyoxalamide Groups in Heterocyclic Bases. J. Bioorganic Chem..

[49] Wang Y., Rösner D., Grzywa M., Marx A. (2014). Chain-Terminating and Clickable NAD+ Analogues for Labeling the Target Proteins of ADP-Ribosyltransferases. Angew. Chem. Int. Ed..

[50] Kovács T., Ötvös L. (1988). Simple synthesis of 5-vinyl- and 5-ethynyl-2′-deoxyuridine-5′-triphosphates. Tetrahedron Lett..

[51] Yoshikawa M., Kato T., Takenishi T. (1967). A novel method for phosphorylation of nucleosides to 5′-nucleotides. Tetrahedron Lett..

[52] Barradas R.G., Fletcher S., Porter J.D. (1976). The hydrolysis of maleimide in alkaline solution. Can. J. Chem..

[53] Sherstyuk Y.V., Ivanisenko N.V., Zakharenko A.L., Sukhanova M.V., Peshkov R.Y., Eltsov I.V., Kutuzov M.M., Kurgina T.A., Belousova E.A., Ivanisenko V.A. (2019). Design, Synthesis and Molecular Modeling Study of Conjugates of ADP and Morpholino Nucleosides as A Novel Class of Inhibitors of PARP-1, PARP-2 and PARP-3. Int. J. Mol. Sci..

[54] Danilin N.A., Koroleva L.S., Novopashina D.S., Venyaminova A.G. (2019). RNase P-Guiding Peptide Conjugates of Oligo(2’-O-methylribonucleotides) as Prospective Antibacterial Agents. Russ. J. Bioorganic Chem..

[55] Mosmann T. (1983). Rapid colorimetric assay for cellular growth and survival–application to proliferation and cyto-toxicity assays. J. Immunol. Methods.

[56] Taskaev S., Berendeev E., Bikchurina M., Bykov T., Kasatov D., Kolesnikov I., Koshkarev A., Makarov A., Ostreinov G., Porosev V. (2021). Neutron Source Based on Vacuum Insulated Tandem Accelerator and Lithium Target. Biology.

[57] Fuentes E., Araya-Maturana R., Urra F.A. (2019). Regulation of mitochondrial function as a promising target in platelet activation-related diseases. Free Radic. Biol. Med..

[58] Peters R.A., Nord F.F. (1957). Mechanism of the toxicity of the active constituent of Dichapetalum cymosum and related compounds. Advances in Enzymology.

[59] Cerqueira N.M., Fernandes P.A., Ramos M.J. (2007). Understanding ribonucleotide reductase inactivation by gemcitabine. Chemistry.

[60] Alvarellos M.L., Lamba J., Sangkuhl K., Thorn C.F., Wang L., Klein D.J., Altman R.B., Klein T.E. (2014). PharmGKB summary: Gemcitabine pathway. Pharm. Genom..

[61] Peters T. (1996). The Albumin molecule: Its structure and chemical properties. All about Albumin: Biochemistry, Genetics, and Molecular Applications.

[62] Sivaev I.B., Kulikova N.Y., Nizhnik E.A., Vichuzhanin M.V., Starikova Z.A., Semioshkin A.A., Bregadze V.I. (2008). Practical synthesis of 1,4-dioxane derivative of the *closo*-dodecaborate anion and its ring opening with acetylenic alkoxides. J Organomet. Chem..

[63] Franken N., Rodermond H., Stap J., Haveman J., van Bree C. (2006). Clonogenic assay of cells in vitro. Nat. Protoc..

