# Supplementary material for: Design of the New Closo-Dodecarborate-Containing Gemcitabine Analogue for the Albumin-Based Theranostics Composition
Source: Molecules. 2023 Mar 15;28(6):2672. doi: 10.3390/molecules28062672 (PMC10056911; doi:10.3390/molecules28062672)
Supplement: Supplementary file 1 [file molecules-28-02672-s001.zip › molecules-2243702-supplementary.pdf]

Supporting Information for

Design of the new closo-dodecarborate-containing gemcitabine analogue for the albumin-based theranostics composition.

Valeria I. Raskolupova [1], Meiling Wang [2], Maya A. Dymova [1], Gleb O. Petrov [1], Ivan M. Shchudlo [3], Sergey Yu. Taskaev [2,3], Tatyana V. Abramova [1], Tatyana S. Godovikova [1,2], Vladimir N. Silnikov [1], Tatyana V. Popova [1,2*].

[1] Institute of Chemical Biology and Fundamental Medicine, SB RAS, 630090 Novosibirsk, Russia; v.raskolupova@mail.ru (V.I.R.); maya.a.rot@gmail.com (M.A.D.); petrov.olegovich@gmail.com (G.O.P.) t_godovikova@mail.ru (T.S.G); abramova@niboch.nsc.ru (T.V.A.); silnik@niboch.nsc.ru (V.N.S.); io197724@gmail.com (T.V.P.)

[2] Novosibirsk State University, Faculty of Natural Sciences, 630090 Novosibirsk, Russia; io197724@gmail.com (T.V.P.); 153890812@qq.com (M.V.); s.yu.taskaev@inp.nsk.su (S.Yu.T.)

[3] Budker Institute of Nuclear Physics, SB RAS, 630090 Novosibirsk, Russia; s.yu.taskaev@inp.nsk.su (S.Yu.T.); cshudlo.i.m@gmail.com (I.M.S.)

* Correspondence: io197724@gmail.ru; Tel.: +8-383-3635183

Spectral data for new compounds

4-N-benzoyl-2′-deoxy-2′,2′-difluorocytidine (2)

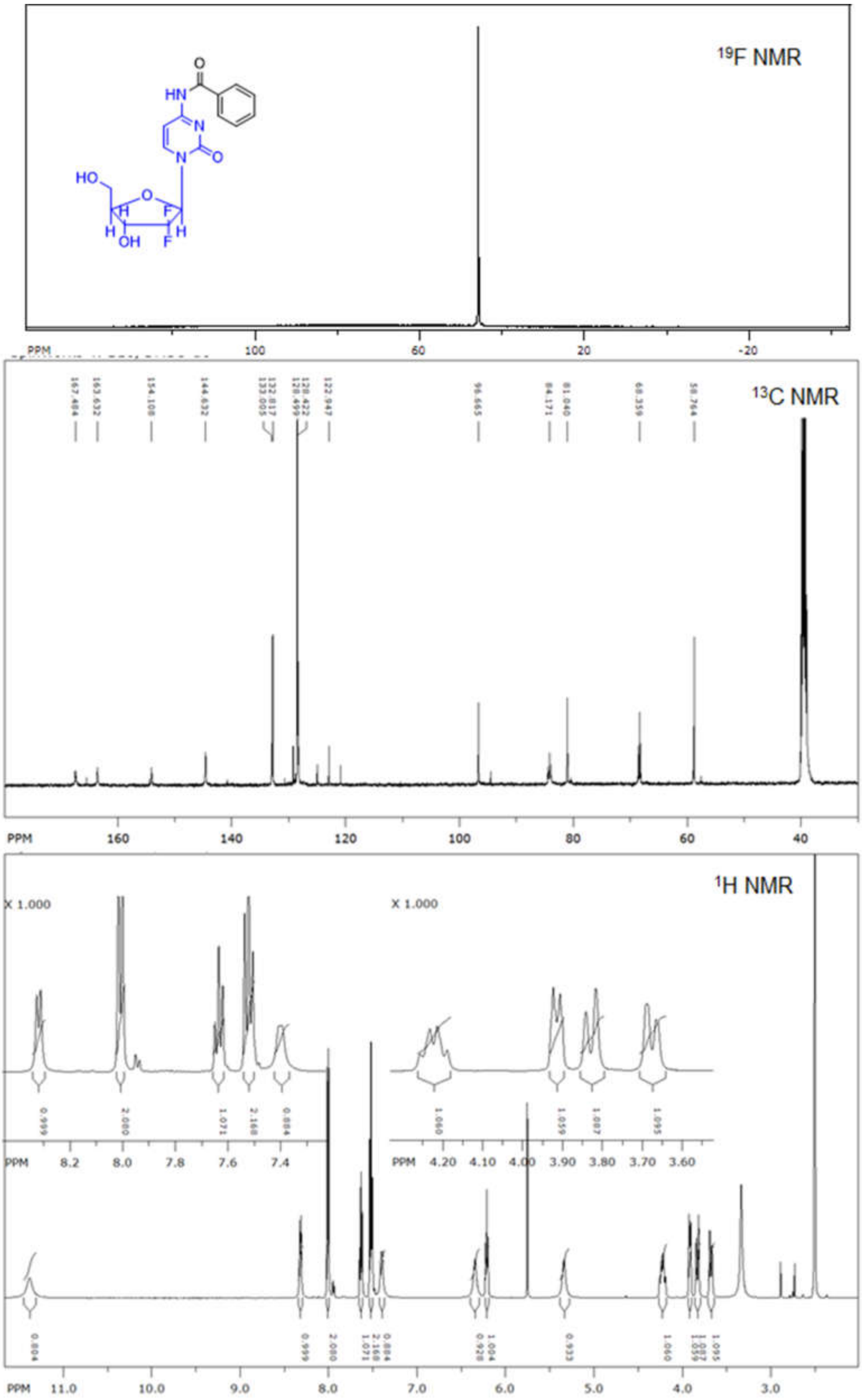

Figure S1. Characterisics of the 4-*N*-benzoyl-2′-deoxy-2′,2′-difluorocytidine.

4-N-(2-dimethylaminoethyl)-2′-deoxy-2′,2′-difluorocytidine (3)

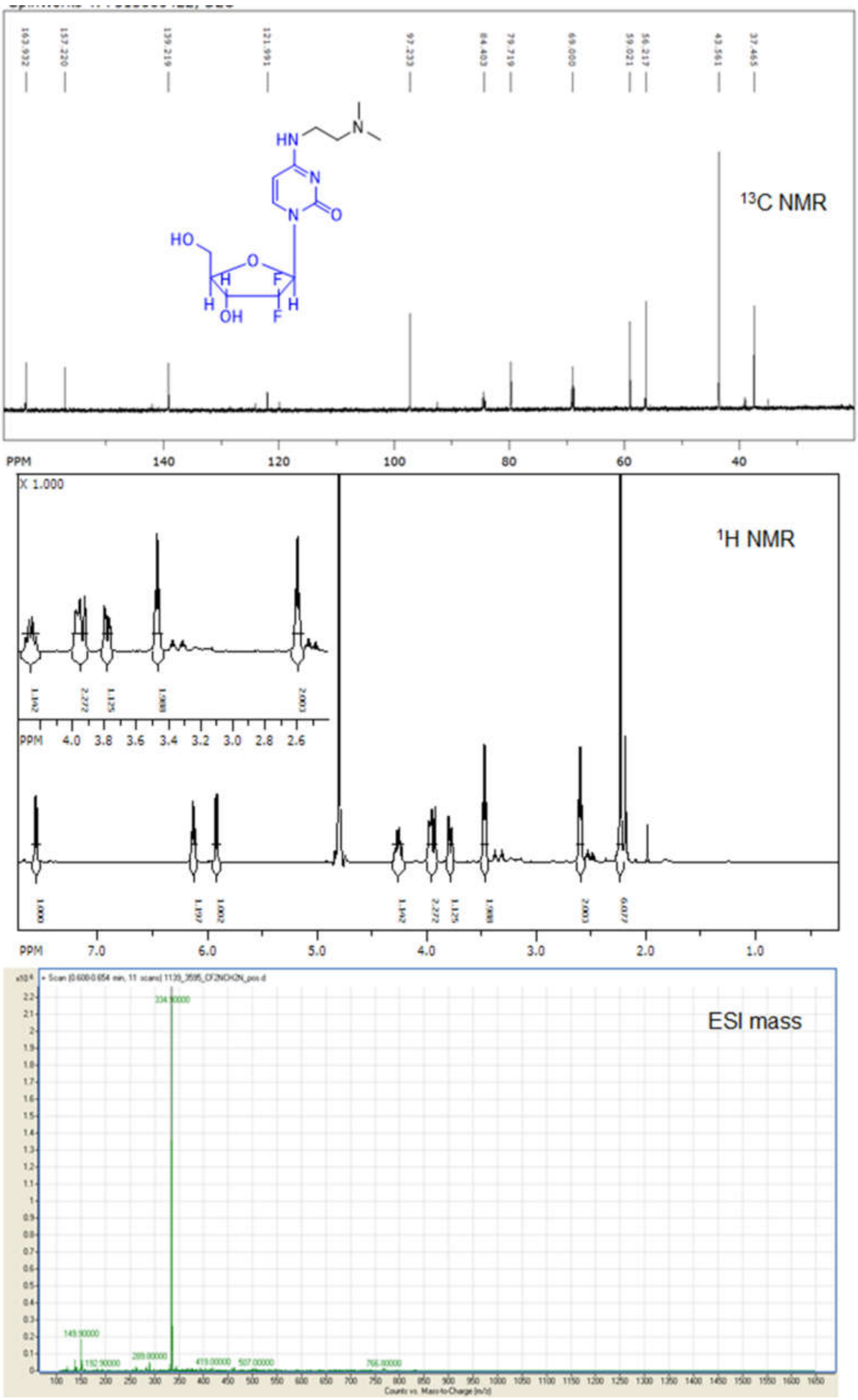

Figure S2. Characterisics of the 4-*N*-(2-dimethylaminoethyl)-2′-deoxy-2′,2′-difluorocytidine

H+[Conjugate 5]-

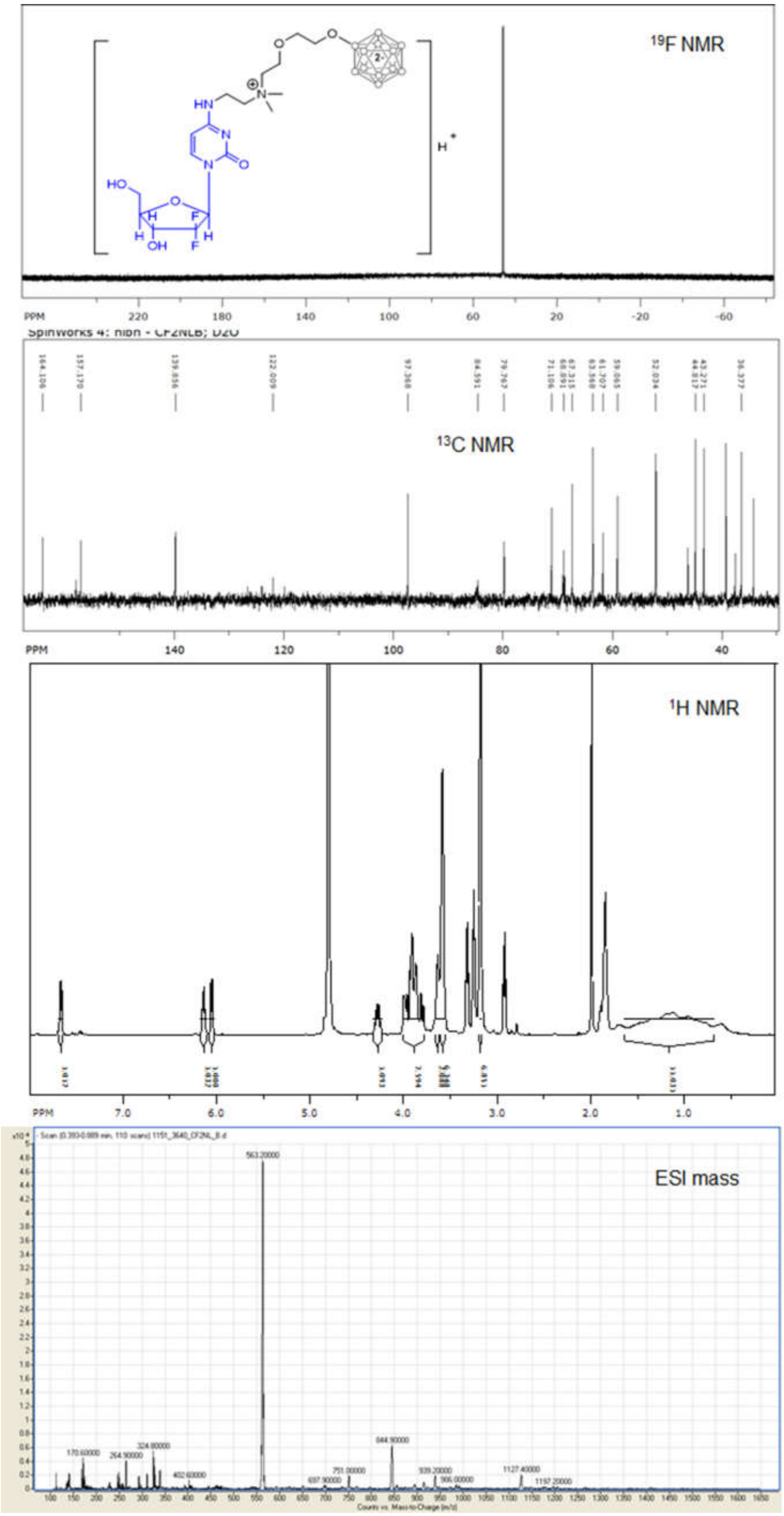

Figure S3. Characterisics of the H+[Compound 5]-.

[Compound 6](TEA)₃

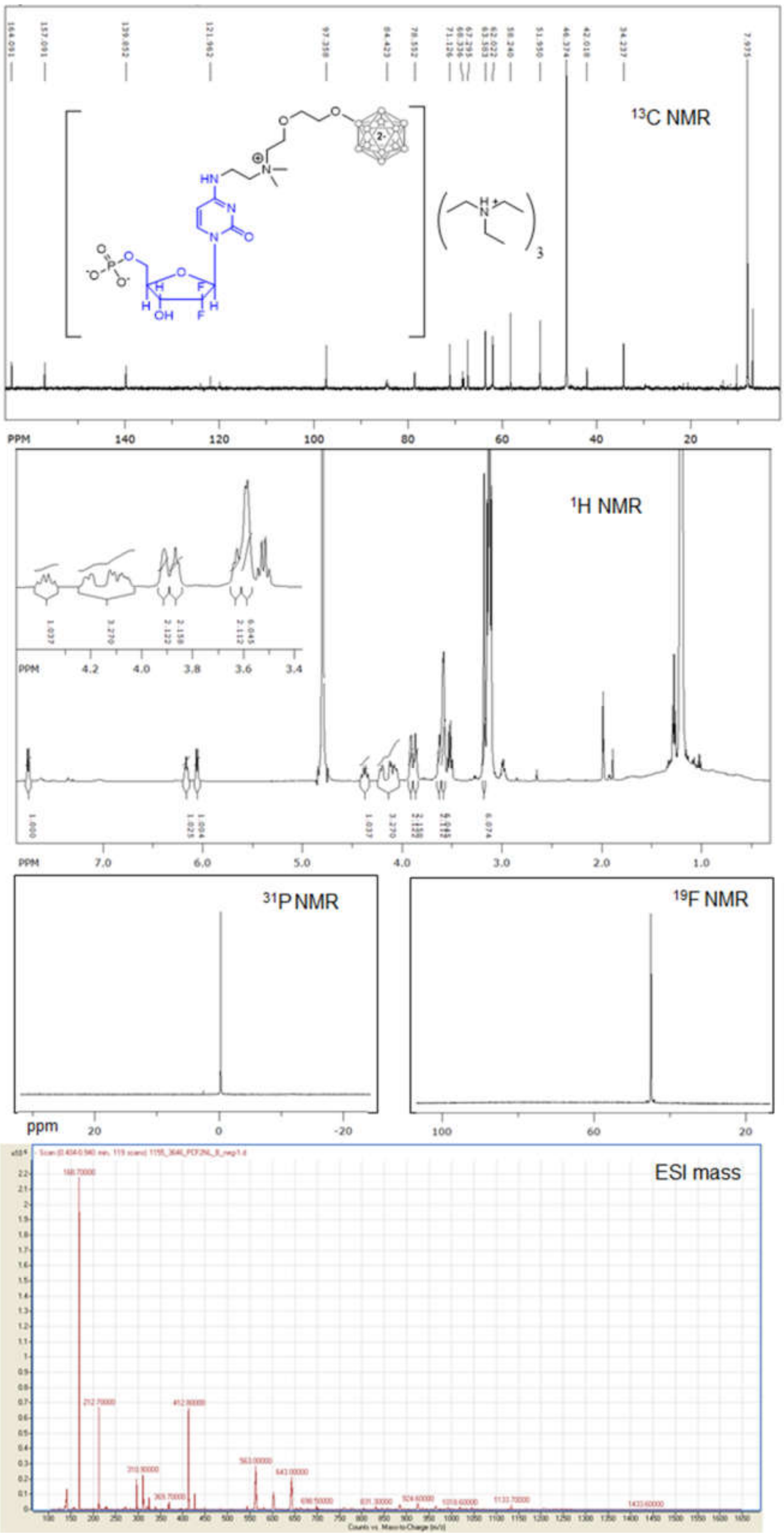

Figure S4. Characterisics of the [Compound 6](TEA)3.

[Compound 7](TEA)$_2$

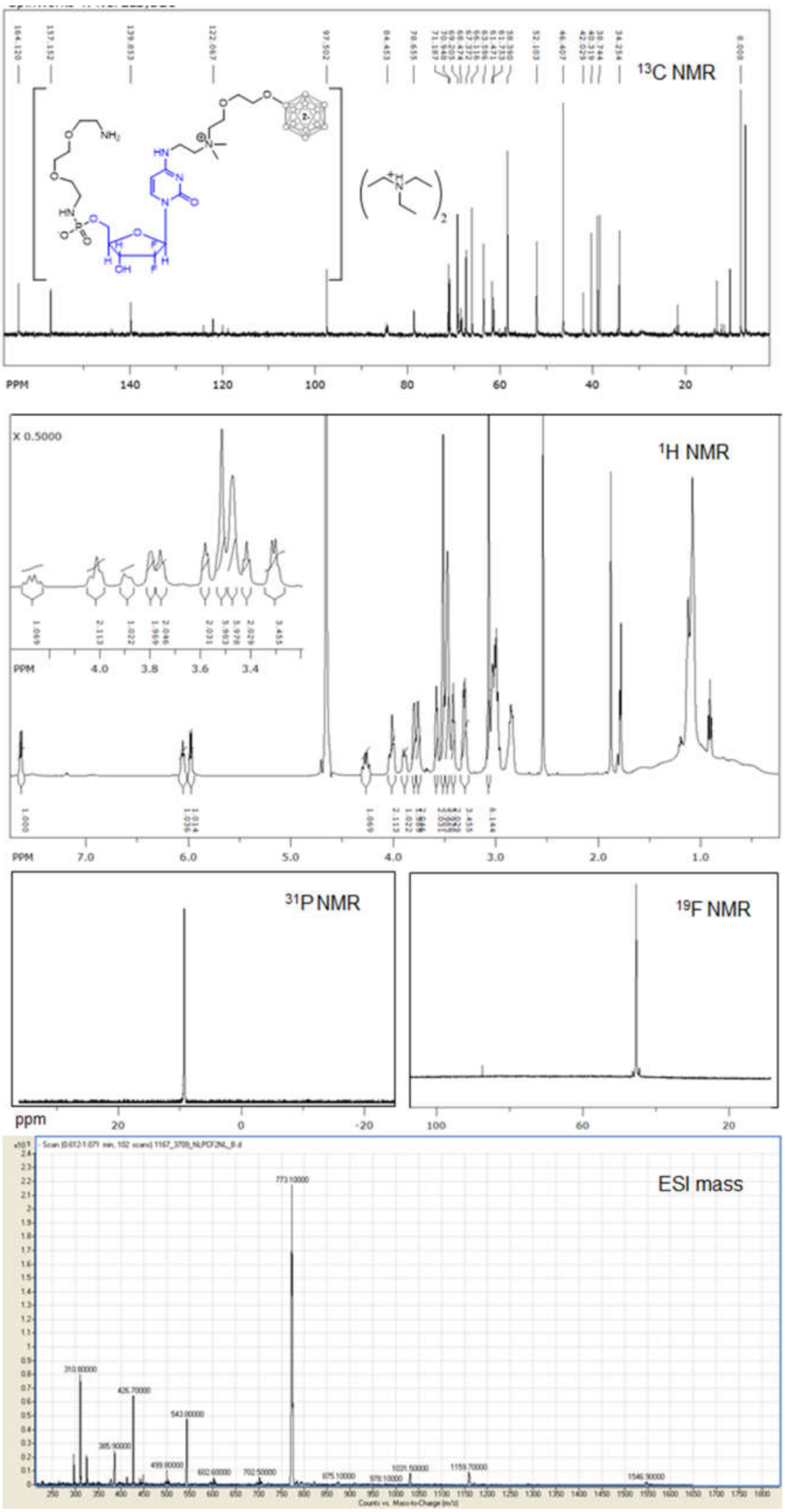

Figure S5. Characterisics of the [Compound 7](TEA)2.

[Compound 1](TEA)₂

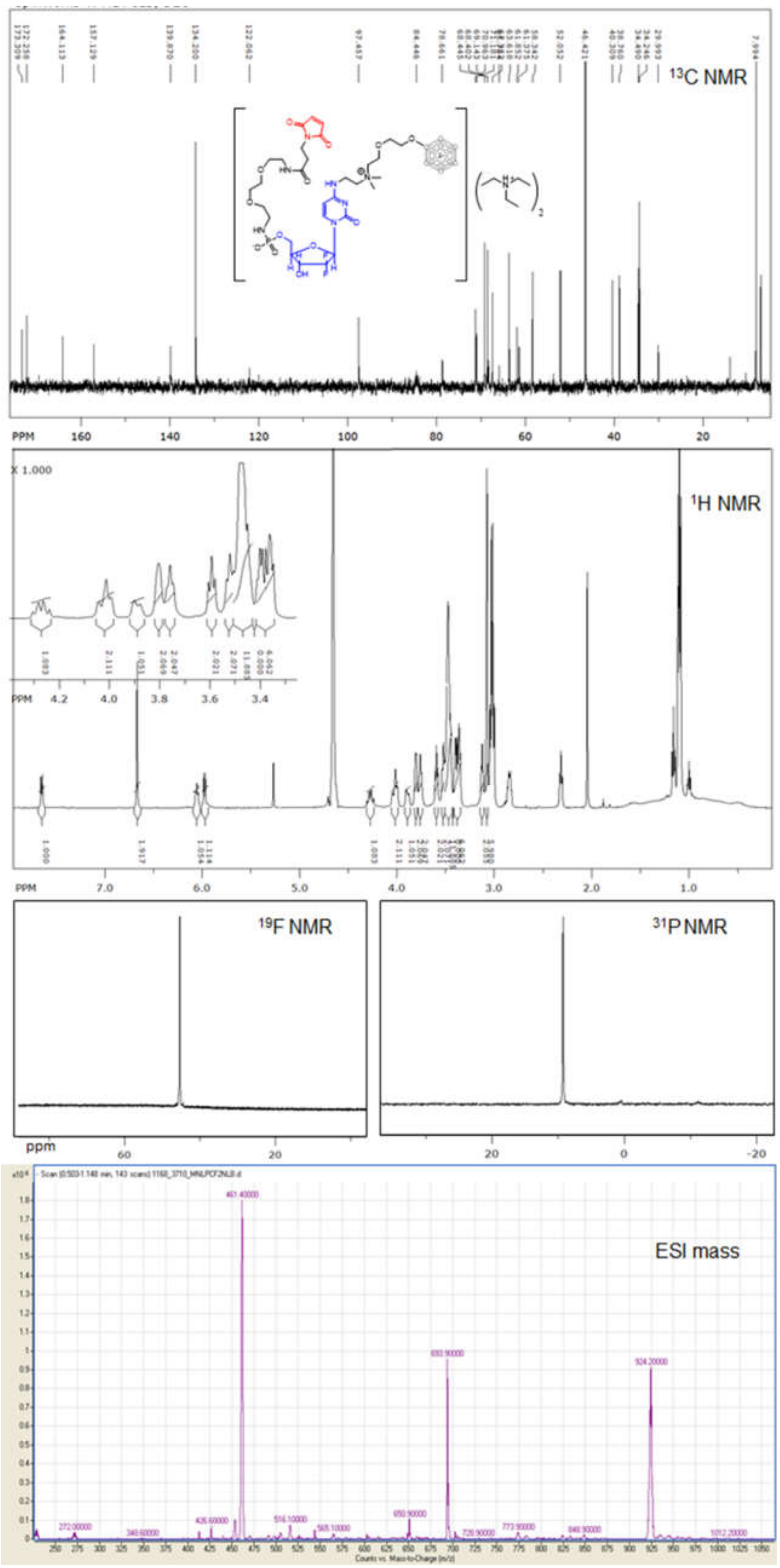

Figure S6. Characterisics of the [Compound 1](TEA)$_2$.

Compound 9

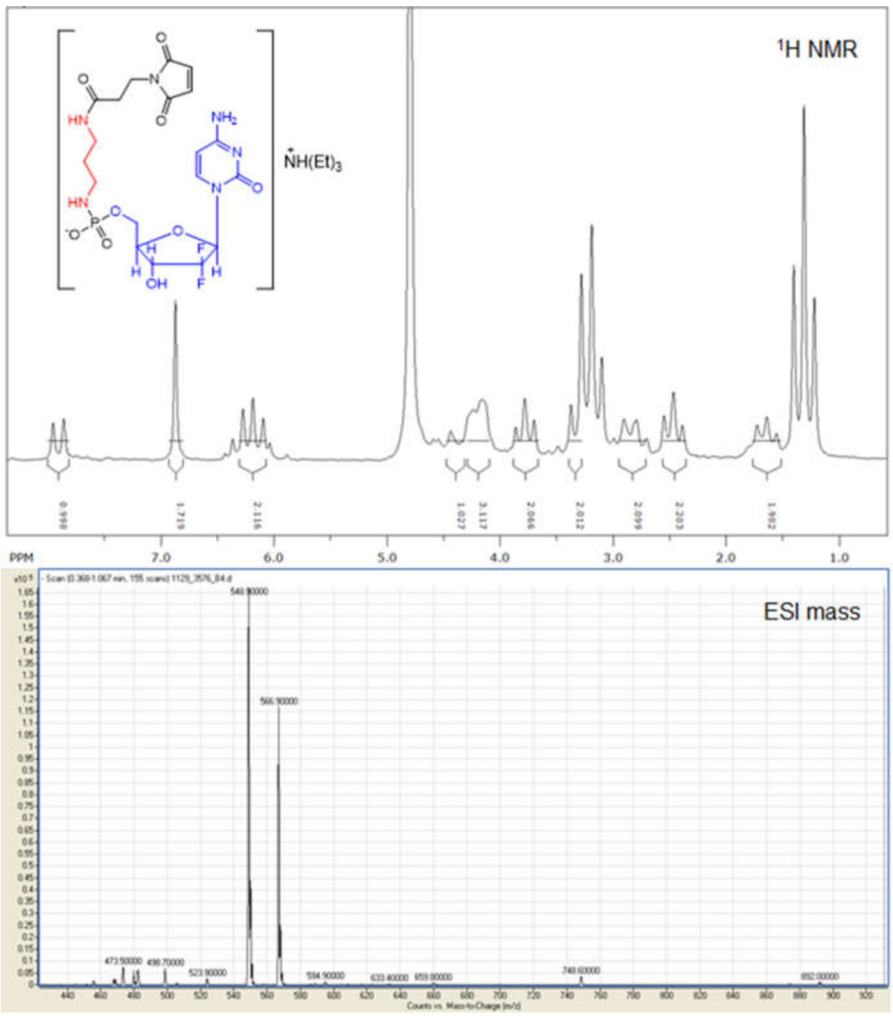

Figure S7. Characterisics of the [Compound 9](TEA).

^{1}H NMR (D_2O): 7.91 (1H, d, *J* 7.6, H6), 6.88 (2H, s, CH=CH), 6.31-6.07 (2H, m, H1', H5), 4.47-4.31 (1H, m, H3'), 4.30-4.09 (3H, m, H4', H5'), 3.78 (2H, t, *J* 6.5, CH2N), 3.39-3.28 (2H, m, CH_2NHCO), 2.95-2.71 (2H, m, CH_2CO), 2.46 (2H, t, *J* 6.5, CH_2NHP), 1.64 (2H, t, *J* 6.8, CH_2). $[M-H]^-$ calcd. for $C_{19}H_{24}F_2N_6O_9P^-$ 549.13; found 548.90.

*Compound 9**

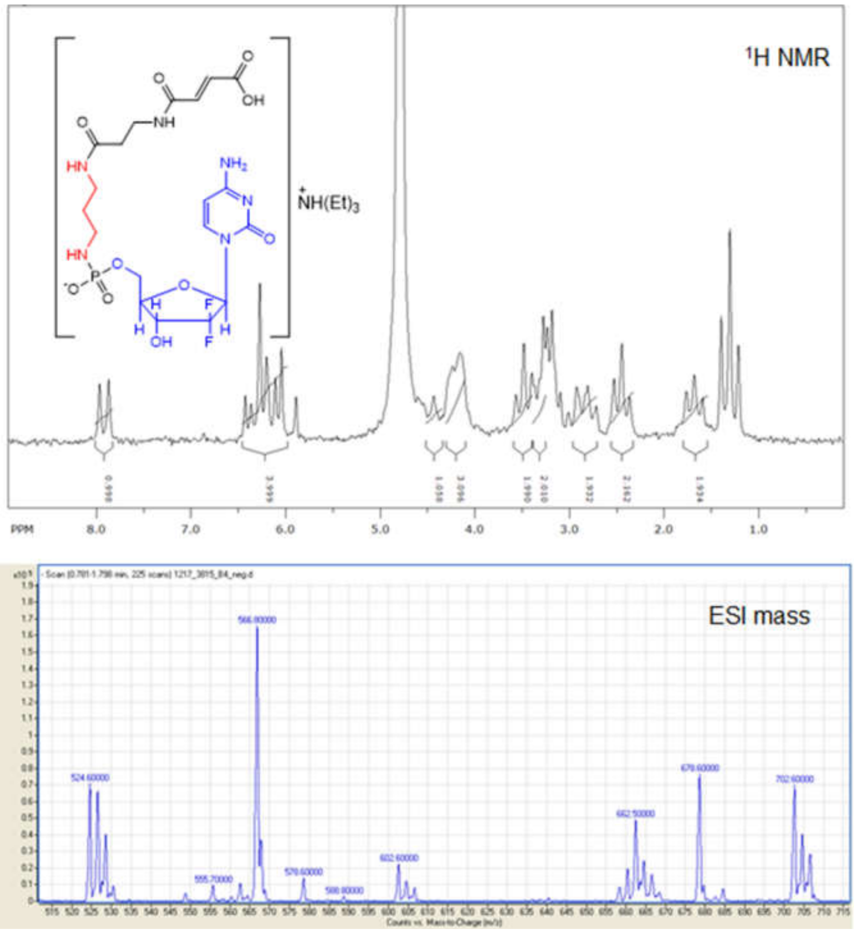

Figure S8. Characterisics of the [Compound 9*](TEA).

^{1}H NMR (D_2O): 7.92 (1H, d, *J* 7.6, H6), 6.46-5.98 (4H, m, CH=CH, H5, H1′), 4.52-4.34 (1H, m, H3′), 4.30-4.10 (3H, m, H4′, H5′), 3.48 (2H, t, *J* 6.7, CH_2NH), 3.38-3.25 (2H, m, CH_2NHCO), 2.96-2.71 (2H, m, CH_2CO), 2.45 (2H, t, *J* 6.7, CH_2NHP), 1.68 (2H, t, *J* 6.8, CH_2). [M-H]$^-$ calcd. for $C_{19}H_{26}F_2N_6O_{10}P^-$ 567.14; found 566.90.

Compound 10

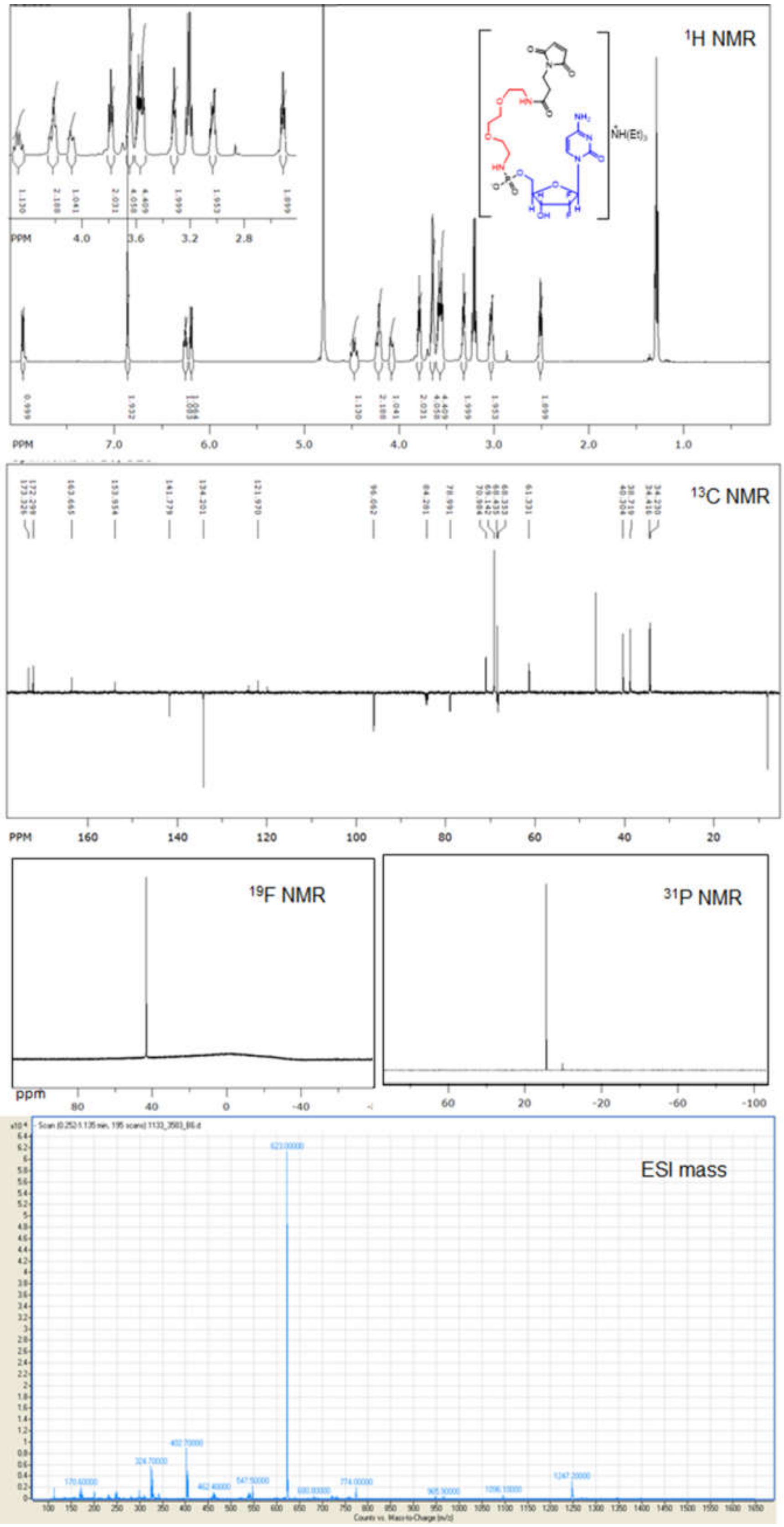

Figure S9. Characterisics of the [Compound 10](TEA).

^1H NMR (D_2O): 7.97 (1H, d, *J* 7.7, H6), 6.86 (2H, s, CH=CH), 6.26 (1H, t, *J* 7.1, H1'), 6.20 (1H, d, *J* 7.7, H5), 4.52-4.43 (1H, m, H3'), 4.26-4.18 (2H, m, H5'), 4.11-4.05 (1H, m, H4'), 3.79 (2H, t, *J* 6.3, CH_2N), 3.67-3.62 (4H, m, CH_2O), 3.58 (2H, t, *J* 5.5, O$\underline{CH_2}$$CH_2$O), 3.55 (2H, t, *J* 5.5, O$CH_2$$\underline{CH_2}$O), 3.32 (2H, br t, *J* 5.3, $\underline{CH_2}$NHCO), 3.05-3.01 (2H, m, $\underline{CH_2}$NHP), 2.51 (2H, t, *J* 6.4, $\underline{CH_2}$CONH). ^{13}C NMR (D_2O): 173.33, 172.30, 163.67, 153.95, 141.78, 134.20, 121.97 (t, J_{CF} 259), 96.06, 84.28 (t, J_{CF} 23), 78.99, 70.98, 69.14, 68.44, 68.35 (t, J_{CF} 23), 61.33, 40.30, 38.72, 34.42, 34.23. ^{19}F NMR (D_2O): 43.14 (br s, CF_2). ^{31}P NMR: 8.67 (s). $[M-H]^-$ calcd. for $C_{22}H_{30}F_2N_6O_{11}P^-$ 623.17; found 623.00.

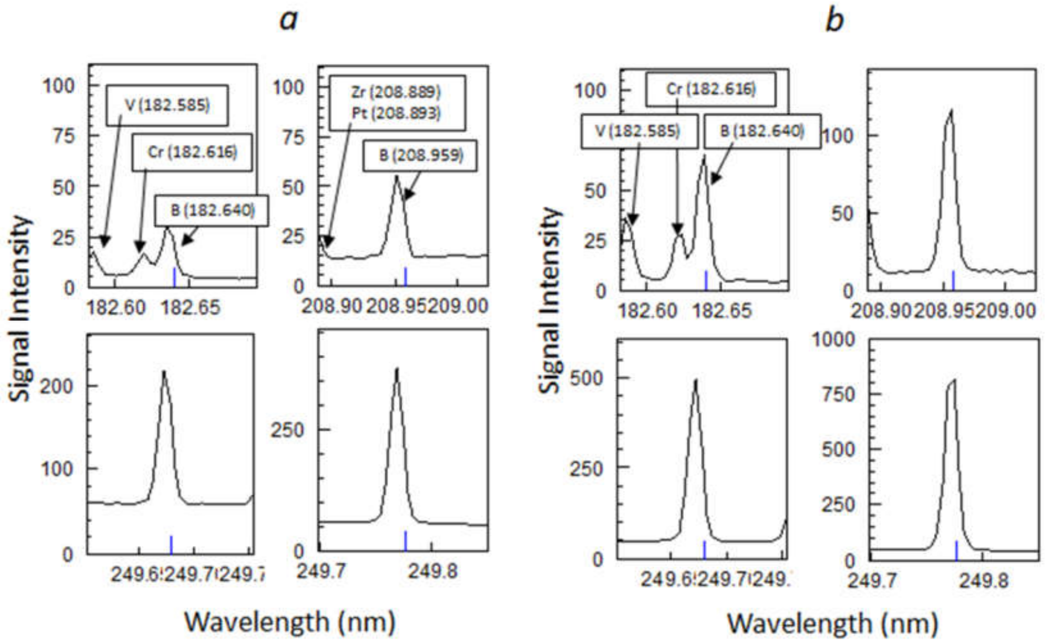

Figure S10. Inductively coupled plasma atomic emission spectrometry (ICP AES) for HSA-Cy5-HcyTFAc-GC$B_{12}H_{11}$ (*a*) and HSA-Cy7-HcyTFAc-GC$B_{12}H_{11}$ (*b*). Boron spectral lines 182.640, 208.959, 249.678 and 249.773 nm.

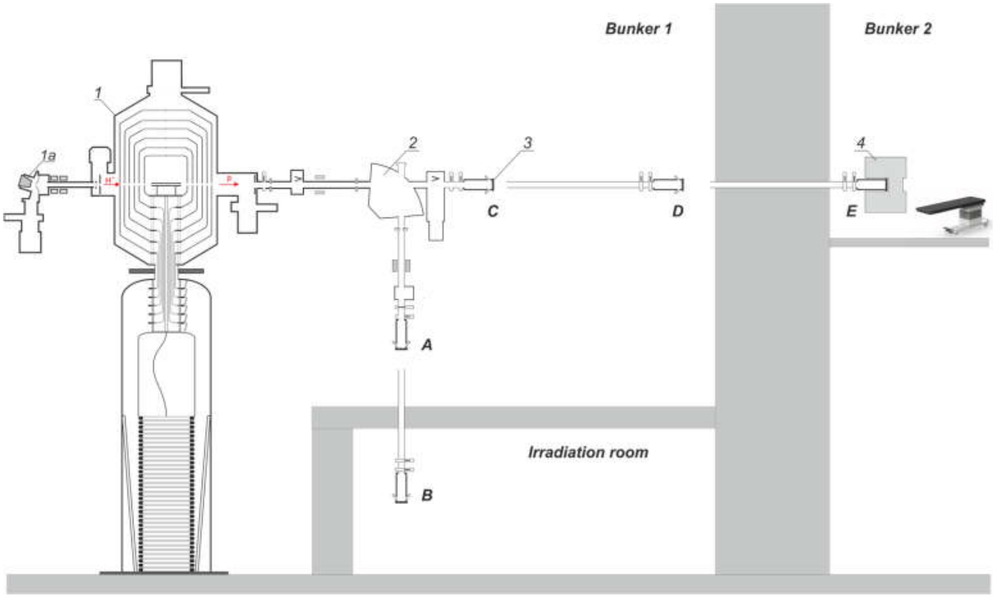

Figure S11. Layout of the experimental facility: *1* – vacuum insulated tandem accelerator, *2* – bending magnet, *3* – lithium target, *4* – beam shaping assembly. *A*, *B*, *C*, *D*, *E* – lithium target placement positions.
